# Psychometric evidence of the Ambivalent Sexism Inventory in undergraduate nursing students at a public institution^*^

**DOI:** 10.1590/1518-8345.7179.4413

**Published:** 2025-01-31

**Authors:** Susane Vasconcelos, Lucia Helena Garcia Penna, Nilton Soares Formiga, Rudson Oliveira Damasceno, Liana Viana Ribeiro, Bianca Carvalho de Abreu Leira

**Affiliations:** 1 Universidade do Estado do Rio de Janeiro, Rio de Janeiro, RJ, Brazil.; 2 Scholarship holder at the Coordenação de Aperfeiçoamento de Pessoal de Nível Superior (CAPES), Brazil.; 3 Universidade Potiguar, Natal, RN, Brazil.; 4 Universidade Estadual do Sudoeste da Bahia, Jequié, BA, Brazil.

**Keywords:** Sexism, Factor Analysis Statistical, Education Nursing, Nursing, Prejudice, Women’s Rights

## Abstract

**Objective:**

to verify the internal and structural consistency of the Ambivalent Sexism Inventory in young undergraduate nursing students.

**Method:**

this is a cross-sectional methodological study carried out with young university students enrolled in the undergraduate Nursing course at a public university. Data was obtained by means of a sociodemographic/academic questionnaire and the Ambivalent Sexism Inventory. The analysis used Pearson’s correlation, Cronbach’s alpha, intraclass correlation, t-test and chi-square ratio and degrees of freedom, as well as confirmatory factor analysis to test the consistency of the existence of the bifactor model.

**Results:**

the sample consisted of 305 undergraduates. The oblique bifactor model showed statistical indicators that justify the consistency of the bifactor structure of sexism in the study’s target population. In addition, the psychometric indicators of the inventory showed satisfactory results. The predictive regression analysis confirmed the structure, demonstrating its consistency and robustness for assessing both hostile sexism and benevolent sexism among young university nursing students.

**Conclusion:**

support for the theory of ambivalent sexism was identified, reflecting the consistency of the oblique bifactor model. The analysis of the psychometric properties of the inventory, including validity and reliability, reinforces its applicability and relevance in research on gender issues in the health area.

## Highlights

**(1)** Phlebitis was the most prevalent complication.

**(2)** Pressure in the bag was associated with catheter removal before indication.

**(3)** Length of stay and use of sedation were related to the onset of phlebitis.

## Introduction

Prejudice associated with gender continues to be a topical issue in the 21^st^ century. Despite scientific progress, it can be seen that the patriarchal construct still produces the cultivation of male domination in social relations, causing women with different characteristics to remain the target of various situations arising from the condition of being a woman, such as the attribution of a passive role, where the process of female submission is gradually considered legitimate, reinforcing socially constructed stereotypes related to sex and gender^(1-2)^.

Stereotypical views of women and men influence the formation and structuring of sexism. The term sexism can be defined as the set of stereotypes relating to appearance, actions, abilities, emotions and the appropriate role in society, which is gendered. Although it also stereotypes men, it often reflects more hostile prejudices against women, manifesting itself institutionally and in interpersonal relationships^(3-4)^. Thus, like the traditional definition of prejudice, sexism is camouflaged in society and in social interactions, not always in a way that is considered negative^(2-5)^.

In the Theory of Ambivalent Sexism (TSA, its acronym in Portuguese), developed to understand the relationship between men and women (cisgender and heterosexual), presented by the authors Glick and Fiske in 1996, sexism is understood in two ways: hostile and benevolent, in any case reflecting the inferiority of women^(6)^. Based on this theory, the same authors developed an instrument capable of identifying and assessing these types of sexism. The so-called Ambivalent Sexism Inventory (ASI), used as the basis for this study, seeks to quantify the expressions of sexism precisely in its bifactor structure (hostile and benevolent sexist attitudes).

Originally developed in English, the ASI initially consisted of 140 items. After adaptations, a reduced version of 22 items was applied to various samples, demonstrating acceptable internal consistency coefficients in the studies. These items make up the final instrument and make it possible to assess the stereotypes assumed by males and females in relation to the two dimensions of sexism^(6)^.

Participants rate the statements on whether they agree or disagree. Items ASI 01, ASI 03, ASI 06, ASI 08, ASI 09, ASI 12, ASI 13, ASI 17, ASI 19, ASI 20 and ASI 22 assess benevolent beliefs, such as “women should be cherished and protected by men” and “man is incomplete without woman”. Meanwhile, items ASI 02, ASI 04, ASI 05, ASI 07, ASI 10, ASI 11, ASI 14, ASI 15, ASI 16, ASI 18 and ASI 21 explore hostile beliefs, such as “women are trying to gain power by controlling men” and “feminist women are making completely unreasonable demands of men”.

Other scales and instruments have been created with the aim of identifying and measuring sexism in different contexts of society, but the ASI was chosen because it has been widely used and validated in various studies around the world, and is recognized as an effective tool for assessing sexist attitudes in different contexts^(7-11)^.

The ASI has revealed psychometric indicators with samples in countries such as the United States, Chile, Mexico, Korea, Germany and Brazil, guaranteeing its bifactor structure attributed to hostile and benevolent sexist attitudes^(7)^. In Brazil, the ASI was adapted and validated by Formiga, Santos and Gouveia^(5)^. The inventory has maintained its psychometric quality over the last few years in samples of men, groups of psychologists, military personnel and in comparisons between Brazilians and Portuguese^(8-11)^, but not yet used in nursing.

For a better understanding, sexism can be compared to an iceberg: where most of it is covered up and only the tip is visible. Hostile sexism would be the visible tip of the iceberg, the most blatant and explicit expression of prejudice in its traditional form: women are considered inferior to men and incapable of performing the same roles as them. It is marked by rejection, antipathy and intolerance towards the female role as a figure of power and decision, belittling women who challenge conservative roles and traditional gender ideologies^(5,12)^.

The covert part of the icerberg is considered the most modern expression of sexism. In a benevolent way, it presents apparently non-prejudiced, subjectively positive actions that define women as a dependent, fragile, sensitive identity that needs support and attention from men. It is also based on the denial of the existence of discrimination against women, thus antagonizing women’s current struggles for greater inclusion in society^(3,13)^.

Everyday sexism is also present in the academic environment. The university is a space for social reproduction, where the same socialization of gender relations takes place as on the “outside”, i.e. outside its walls. And even though it is considered a space for plurality of thought, violent attitudes of discrimination and intolerance can multiply in it. In addition, this environment maintains strong hierarchical structures that favor unequal relations^(14)^.

The training and development of nursing students as professionals depends on relational and environmental characteristics^(12)^. Nursing is considered a historically female profession, due to its characteristics and the prevalence of women, who find gender inequality an indicator of greater vulnerability. Unfair and avoidable sexist experiences must be studied in order to better educate young people and consequently have a positive influence on their future practices. The scarcity of studies on the use of the Ambivalent Sexism Inventory in the population of young nursing undergraduates means that there are still gaps in our knowledge about inequality and sexism in this context. Studies aimed at researching and exploring sexism in the university environment and in nursing courses are needed.

Thus, this study aims to verify the internal and structural consistency of the Ambivalent Sexism Inventory in young undergraduate nursing students.

## Method

### Study design

This is a cross-sectional methodological study with a quantitative approach, written in accordance with the STROBE tool (Strengthening the Reporting of Observational Studies in Epidemiology).

### Study setting

The study was carried out at a public Higher Education Institution (HEI) in Rio de Janeiro - RJ, Brazil: Universidade do Estado do Rio de Janeiro. The HEI was chosen because of its renowned reputation in the field of nursing and because it is an internationally recognized research center.

### Population and sample

The target population for this study was made up of undergraduate nursing students enrolled between July and October 2022, totaling 352 students. All students who met the inclusion criteria - over 18 years old and actively enrolled - were invited to take part in the study. After applying the exclusion criteria, which involved the student not being found after three attempts, the final sample consisted of 305 participants. In order to determine the sample size, the recommendations recommended in the literature for confirmatory factor analysis were taken into account, which suggest the participation of at least 200 to 300 participants to ensure the robustness of the model parameter estimates^(15)^. Thus, the number of 305 participants was considered adequate for the analysis.

### Data collection

Data was collected between June and October 2022. The data was obtained using a self-administered instrument. Initially, the nominal list of students enrolled by term and the timetables of the subjects was made available by the course secretary. Then, after contacting the teacher responsible for the selected subject, the survey was presented in a standardized way and the instrument was distributed in the classroom. Students who met the inclusion criteria were invited to take part and fill in the questionnaire individually. The nursing students took an average of 30 minutes to complete the questions.

The data collection instrument was divided into two blocks: block I covered sociodemographic aspects such as gender, age, marital status, gender identity and expression, sexual orientation, occupation, religion, family income, housing and leisure. Block II consisted of the Ambivalent Sexism Inventory - ASI^(5-6)^.

The inventory, being self-administered, allowed students to indicate how much they agreed with the content expressed, using a four-point scale with the following possible answers: 1 = Strongly disagree; 2 = Disagree; 3 = Agree and 4 = Strongly agree.

No items were identified that generated difficulties in understanding on the part of the nursing students during the data collection process.

### Data processing and analysis

The data was organized, tabulated and analyzed using the Statistical Package for the Social Sciences (SPSS) software, version 25.0. The descriptive analysis of the population’s characteristics was expressed through frequencies (absolute and relative) for the categorical variables, and for the quantitative variables we used measures of central tendency (mean, median) and dispersion (standard deviation) and calculations relating to Pearson’s correlation, Cronbach’s alpha (α), intraclass correlation and chi-square ratio and degrees of freedom.

In order to verify the relationship between the content of the 22 ASI items, based on their behavior-domain representativeness, correlational analysis was used to assess the theoretical relationship presented in the instrument developed by the aforementioned authors, as well as the situations specified in the items and the extent to which this instrument represents the expected aspects. To this end, correlational analysis was used, calculating Pearson’s correlation coefficient.

Having theoretically assumed that the content of the sexism items discriminate and represent this concept and the theoretical-empirical proposal of the construct’s bifactoriality (i.e. ambivalent sexism, structured into benevolent and hostile sexism), the Amos Graphics 24.0 statistical package was used for the confirmatory factor analysis, which hypothesized the bifactorial model observed by the aforementioned authors, from which it was expected that the item-factor organization would confirm a similar association already observed.

Confirmatory factor analysis was used to test the consistency of the existence of the bifactor model of sexism (hostile and benevolent) in undergraduate nursing students. We chose to leave the covariances (phi, φ) free, revealing indicators of quality of fit for the proposed model that are close to the recommendations presented in the literature^(15-16)^.

In this way, the ML (Maximum Likelihood) estimator was taken as the input for the covariance matrix. As this is a more careful and rigorous type of statistical analysis, we sought to evaluate the theoretical structure of the ASI, using the Glick and Fiske^(6)^ and Formiga, Santos and Gouveia^(5)^ proposals for bifactoriality as an empirical and axiomatic guideline. As far as this analysis is concerned, the following statistical indicators were taken into account to assess factor quality: chi-square/degrees of freedom (χ²/gl); residual root mean square (RMR); goodness-of-fit index (GFI) and adjusted goodness-of-fit index (AGFI); comparative fit index (CFI); Tucker-Lewis index (TLI); root-mean-square error of approximation (RMSEA); expected cross-validation index (ECVI) and consistent Akaike information criterion (CAIC); Akaike›s information criterion (AIC); Browne-Cudeck criterion (BCC); Bayes information criterion (BIC); calculation of composite reliability (CR) and average variance extracted (AVE); Cronbach’s alpha (α): Fornell and Larcker criterion and AVE; interclass correlation coefficient (ICC).

During data processing and analysis, statistical tests were used in an integrated manner to investigate the psychometric properties of the ASI items. This approach allowed not only an analysis of the bifactor structure of sexism, as proposed by the theory underlying the instrument, but also empirically validated its consistency.

### Ethical aspects

This study complied with the ethical principles proposed by Resolution 674/22 of the National Health Council. Certificate of Submission for Ethical Appraisal (CAAE): 59881522.0.0000.5282.

## Results

The population was composed of 305 students, most were females, with a total of 266 students, 87.2% of the population. The predominant age group was between 20-24 years (n=211; 69.2%), with an average age of 23 years old (standard deviation 4.5), with the youngest participant being 18 years old and the oldest 56 years old. With regard to color/race, blacks (n=64; 21.0%) and browns (n=94; 30.8%) were identified, while whites accounted for 47.9% (n=146). Most young people were single (n=281; 92.1%), with a family income of between one and three minimum wages (n=183; 60%).

To ensure the quality of the sample, the multicollinearity criterion was included in the evaluation. It was observed that the correlations between the Tabachnick and Fidell parameters had a value of r ≤ 90, ranging from -0.08 to 0.48, indicating the absence of variables with a high degree of correlation, thus allowing the development of predictive and/or correlational models with low measurement error.

Table 1 shows that the students were able to identify and evaluate sexism in the context in question, showing that all the items were significant in their discrimination. Pearson’s correlation calculation (r) for the 22 items revealed strong, positive correlations with the general sexism construct (total score), all of which were significant. This ensured the conceptual and empirical direction of the construct, not excluding any item from the intended measure.

The confirmatory factor analysis (CFA) revealed that the oblique bifactor model (where the factors correlate) showed statistical indicators that justify the consistency of the bifactor structure of sexism in the study’s target population, hostile and benevolent, as shown in Table 2.

Also, when looking at the statistical indicators of comparison that suggest a parsimonious evaluation (AIC, BIC and BCC) of the intended factorial model in relation to the hypothesized one, it can be seen that the expected bifactor model had strong psychometric indicators, as it follows the CAIC and ECVI, aimed at verifying the adequacy of the structural model (Table 3).

**Table 1:** - Discriminant analysis, content representativeness and item discrimination of the Ambivalent Sexism Inventory in undergraduate nursing students (n = 305). Rio de Janeiro, RJ, Brazil, 2022

**Variables**	**Minimum**	**Maximum**	**Mean**	SD*	**Sk** ^†^	**Ku** ^‡^	**Representativeness and breakdown of items**	
							**r** ^§^ **(≥ 0.50)**	**t** ^||^ **(≥ 1.96)**
ASI^¶^01	1	4	1.34	0.67	2.19	1.63	0.58^§^	-7.68^||^
ASI^¶^02	1	4	1.41	0.76	1.88	1.74	0.50^§^	-8.04^||^
ASI^¶^03	1	4	1.96	1.01	0.62	0.85	0.50^§^	-8.96^||^
ASI^¶^04	1	4	1.50	0.77	1.43	1.22	0.52^§^	-6.33^||^
ASI^¶^05	1	4	1.30	0.59	1.09	4.35	0.56^§^	-8.82^||^
ASI^¶^06	1	4	1.16	0.43	1.20	1.98	0.58^§^	-6.68^||^
ASI^¶^07	1	4	1.25	0.57	1.62	1.29	0.59^§^	-7.61^||^
ASI^¶^08	1	4	1.48	0.77	1.55	1.56	0.64^§^	-9.04^||^
ASI^¶^09	1	4	1.81	1.01	0.79	0.79	0.62^§^	-10.33^||^
ASI^¶^10	1	4	1.25	0.51	1.13	1.53	0.60^§^	-6.95^||^
ASI^¶^11	1	4	1.24	0.56	1.74	1.03	0.53^§^	-6.99^||^
ASI^¶^12	1	4	1.45	0.75	1.76	1.37	0.66^§^	-10.42^||^
ASI^¶^13	1	4	1.29	0.59	1.32	1.65	0.65^§^	-9.07^||^
ASI^¶^14	1	4	1.22	0.56	1.90	1.60	0.53^§^	-6.29^||^
ASI^¶^15	1	4	1.24	0.55	1.36	1.95	0.67^§^	-7.28^||^
ASI^¶^16	1	4	1.23	0.53	1.54	1.65	0.57^§^	-6.59^||^
ASI^¶^17	1	4	1.87	1.10	0.85	0.75	0.50^§^	-9.09^||^
ASI^¶^18	1	4	1.40	0.78	1.91	1.67	0.53^§^	-8.15^||^
ASI^¶^19	1	4	1.98	1.06	0.54	1.11	0.55^§^	-11.02^||^
ASI^¶^20	1	4	1.48	0.77	1.62	2.14	0.58^§^	-9.61^||^
ASI^¶^21	1	4	1.18	0.48	1.24	1.36	0.56^§^	-7.18^||^
ASI^¶^22	1	4	2.09	1.10	0.47	1.19	0.52^§^	-8.77^||^

*SD = Standard Deviation; ^†^Sk = Symmetry index; ^‡^Ku = Kurtosis index; ^§^r = Pearson’s correlation (p-value < 0.01); ^||^t = t-test (p-value < 0.01); ^¶^ASI = Items from the Ambivalent Sexism Inventory

**Table 2:** - Psychometric indicators of the factor structure of the Ambivalent Sexism Inventory in undergraduate nursing students (n = 305). Rio de Janeiro, RJ, Brazil, 2022

**Model**	**Measures of fit absolute**				**Incremental adjustment measures**			**Parsimonious adjustment measures**	
Statistics	χ²/gl*	RMR^†^	GFI^‡^	AGFI^§^	CFI^‖^	TLI^¶^	RMSEA** (Intervalo)	CAIC^††^	ECVI^‡‡^ (Interval)
Model1^§§^	2.85	0.06	0.84	0.79	0.84	0.81	0.08 (0.07-0.09)	940.99	2.21 (1.99-2.45)
Model2^‖‖^	2.53	0.09	0.88	0.85	0.87	0.84	0.07 (0.06-0.08)	889.09	2.01 (1.81-2.23)
Model3^¶¶^	1.51	0.03	0.93	0.91	0.95	0.96	0.04 (0.03-0.05)	736.25	1.37 (1.23-1.53)

*χ²/gl = Chi-square ratio/degrees of freedom; ^†^RMR = Residual root mean square; ^‡^GFI = Goodness-of-fit index; ^§^AGFI = Adjusted goodness-of-fit index; ^‖^CFI = Comparative fit index; ^¶^TLI = Tucker-Lewis index; **RMSEA = Root Mean Square Error of Approximation; ^††^CAIC = Consistent Akaike Information Criterion; ^‡‡^ECVI = Expected Cross Validation Index; ^§§^Model1 = Single Factor Model; ^‖‖^Model2 = Orthogonal Bifactor Model; ^¶¶^Model3 = Adjusted Oblique Bifactor Model

**Table 3:** - Psychometric indicators of parsimony for comparing the factor-conceptual structure of the Ambivalent Sexism Inventory in undergraduate nursing students (n = 305). Rio de Janeiro, RJ, Brazil, 2022

	**Parsimony indicator**		
**Model**	AIC*	**BIC** ^†^	**BCC** ^‡^
Model1^§^	671.94	883.99	681.27
Model2^‖^	610.59	830.09	620.25
Model3^¶^	415.27	668.26	426.42

*AIC = Akaike’s information criteria; ^†^BIC = Bayes information criterion; ^‡^BCC = Browne-Cudeck criterion; ^§^Model1 = Unifatorial model; ^‖^Model2 = Orthogonal bifatorial model; ^¶^Model3 = Fitted oblique bifatorial model

All the saturations (Lambdas, λ) were within the expected range |0 - 1|, which revealed that there was no problem with the proposed estimation of the factoriality of sexism (Table 4). In addition, they were statistically different from zero (t > 1.96, p < 0.05) proving the existence of the oblique bifactor model, revealing a positive and strong Phi (φ) association between the hostile and benevolent sexism factors of 0.68. In this condition, it is likely that students who score higher in one dimension will also score higher in the other dimension (Tables 3 and 4).

It should also be noted that the indicators of composite reliability (CR) and average variance extracted (AVE), which refer to the validity of the construct, showed values equal to or higher than those required in the literature^(15-16)^. Specifically, for the hostile sexism dimension, the CR and AVE values were 0.90 and 0.50, respectively. For benevolent sexism, the values were 0.81 and 0.52. These results show the reliability and convergent validity of the construct, demonstrating that the bifactor structure is appropriate in a sample of young nursing undergraduates.

Intraclass correlation was also used to provide greater psychometric assurance of these alphas. The alphas for the psychological measures used were consistent and guaranteed the evaluation of the constructs for these young people. Cronbach’s alpha coefficient (α) presented scores above expectations and was significant. Attention should be drawn to the results of the confidence interval in the intraclass correlation coefficient (ICC), which were in intervals close to those observed in Cronbach’s alpha (α), a condition which guarantees the reliability of the measure in the sample evaluated.

**Table 4:** - Factor structure of the Ambivalent Sexism Inventory in undergraduate nursing students (n = 305). Rio de Janeiro, RJ, Brazil, 2022

ξ*	**χ** ^†^	λ^‡^	Ɛ^§^	**CC** ^‖^	**VME** ^¶^	α**	**ICC** ^††^	**Fornell and Larcker criteria**
	HS11	0.70	0.35				0.85 (0.82-0.87)	
	HS15	0.71	0.26					
	HS5	0.69	0.45					
	HS10	0.70	0.49	0.90	0.50	0.86		0.71
	HS16	0.72	0.52					
	HS21	0.75	0.54					
Hostile sexism (HS)	HS7	0.68	0.46					
⭡	HS4	0.72	0.52					
	HS18	0.77	0.47					
	HS14	0.71	0.37					
	HS2	0.63	0.43					
Φ^‡‡^= 0,68	BS1	0.63	0.40					
⭣	BS6	0.50	0.25					
	BS12	0.74	0.64					
	BS13	0.70	0.50					
	BS3	0.74	0.16				0.82 (0.79-0.85)	
Benevolent sexism (BS)	BS9	0.71	0.38	0.81	0.52	0.82		0.72
	BS17	0.78	0.23					
	BS20	0.66	0.31					
	BS8	0.82	0.38					
	BS19	0.74	0.19					
	BS22	0.66	0.22					

*ξ = Psychological construct; ^†^χ = Variables (items); ^‡^λ = Factor scores of the structure; ^§^ε = Measurement errors of the structure; ^‖^CR = Composite reliability; ^¶^AVE = Average variance extracted; **α = Cronbach’s alpha; ^††^ICC = Intraclass correlation; ^‡‡^φ = Phi

Table 5 shows the result of the bifactor structure being confirmed on the basis of the predictive estimates from the regression analysis. Thus, the proposed model, based on the identification of the variables, presented a ratio/criterion that not only corresponded to what was expected statistically, but was also different from zero (t > 1.96, p < 0.05), with all of them being significant.

**Table 5:** - Indicators of predictive estimates between item-factors of the Ambivalent Sexism Inventory in undergraduate nursing students (n = 305). Rio de Janeiro, RJ, Brazil, 2022

**Variables**	**Relation**	**Constructs**	**Estimate**	SD*	**Ratio/criterion**	**p-value**
ASI^†^11	<---^‡^	HS^§^	1.000	---	---	---
ASI^†^15	<---^‡^	HS^§^	1.253	0.121	10.322	0.001
ASI^†^5	<---^‡^	HS^§^	1.051	0.120	8.736	0.001
ASI^†^10	<---^‡^	HS^§^	1.079	0.109	9.922	0.001
ASI^†^16	<---^‡^	HS^§^	0.997	0.110	9.035	0.001
ASI^†^21	<---^‡^	HS^§^	0.838	0.097	8.635	0.001
ASI^†^7	<---^‡^	HS^§^	1.033	0.126	8.192	0.001
ASI^†^4	<---^‡^	HS^§^	0.915	0.146	6.273	0.001
ASI^†^18	<---^‡^	HS^§^	1.225	0.152	8.049	0.001
ASI^†^14	<---^‡^	HS^§^	0.818	0.109	7.475	0.001
ASI^†^2	<---^‡^	HS^§^	1.071	0.149	7.193	0.001
ASI^†^1	<---^‡^	BS^‖^	1.000	---	---	---
ASI^†^6	<---^‡^	BS^‖^	0.486	0.061	7.926	0.001
ASI^†^12	<---^‡^	BS^‖^	1.306	0.117	11.177	0.001
ASI^†^13	<---^‡^	BS^‖^	0.927	0.088	10.527	0.001
ASI^†^3	<---^‡^	BS^‖^	0.823	0.141	5.854	0.001
ASI^†^9	<---^‡^	BS^‖^	1.347	0.161	8.395	0.001
ASI^†^17	<---^‡^	BS^‖^	1.175	0.155	7.585	0.001
ASI^†^20	<---^‡^	BS^‖^	0.949	0.109	8.667	0.001
ASI^†^8	<---^‡^	BS^‖^	0.999	0.112	8.923	0.001
ASI^†^19	<---^‡^	BS^‖^	0.962	0.148	6.492	0.001
ASI^†^22	<---^‡^	BS^‖^	1.065	0.155	6.854	0.001

*SD = Standard deviation; ^†^ASI = Items from the Ambivalent Sexism Inventory; ^‡^<--- = Direction of association between the construct and Inventory items; ^§^HS = Hostile sexism; ^‖^BS= Benevolent sexism

Statistical analyses were selected based on the objectives of the study and the nature of the data collected. The analyses were essential to evaluate the behavior-domain representativeness of the instrument’s items and to test the consistency of the bifactor model of sexism in undergraduate nursing students, verifying the adequacy of the ASI’s theoretical structure to the data collected.

## Discussion

The results of this research confirm the internal and structural consistency of the Ambivalent Sexism Inventory applied to Nursing undergraduates, supporting the hypothesis of the two-factor model of sexism (hostile and benevolent) proposed by the authors Glick and Fiske^(6)^. These findings reinforce the validity of the instrument while expanding the understanding of the presence and nature of hostile and benevolent sexism in this population. Such evidence is essential to assess how sexist attitudes manifest in the academic environment and their implications for the training of future professionals.

The inventory developed by the authors^(6)^ has been used in other contexts and populations, presenting factorial accuracy close to the original instrument^(8-11,17)^.

As well as representing the proposed content of ambivalent sexism very well, with alpha and ICC scores above the minimum psychometric standard required, the ASI showed consistent applicability in the sample collected. With this result, it is possible to highlight that the organization of the inventory items corresponded to those already found in previous studies, and in relation to the internal consistency of the inventory, the results were better when compared to those previously found^(5,18)^.

As a result, the nursing students who took part in the study were able to recognize the content (sexism) and the meaning of the inventory presented to them. Moreover, each individual item that makes up the ASI is related to the inventory as a whole. The psychometric results suggest that the Brazilian version of the ASI accurately assesses ambivalent sexism, according to the construct used in the instrument’s theoretical model.

There is a consensus that the vast majority of cultures in the world are part of a patriarchal system, which grants power and privileges to the male figure, ensuring male superiority and sovereignty in social relations^(2,19)^. Young people who are part of society and this system think and act influenced by the context in which they live, so even if they don’t mean to, they are influenced and deal with the consequences of sexism, prejudice and violence, and are able to identify situations where gender-related prejudice also occurs in the academic environment^(3,20)^.

Sexism is a subject that needs to be studied, mainly because of its ability to adapt and camouflage itself in society^(9)^. The ambivalent manifestation of sexism among the students also denounces a prejudice against women that at times tries to camouflage itself in apparently non-prejudiced situations, which probably takes root early on in people’s attitudes and thoughts. This seems to confirm the obvious within the Brazilian context, which at first denies the existence of discrimination against women. But in the subtlety, in the praise, in the idea of protection, support and gratitude, it manifests sexism, and thus, between the lines, legitimizes violence against women^(21-22)^.

In addition, the indicators for the absolute adjustment measures: φ.²/gl = 1.51, RMR = 0.03, GFI = 0.93, AGFI = 0.91; incremental adjustment measure: CFI = 0.95, TLI = 0.96, RMSEA = 0.04 (0.03-0.05); parsimonious adjustment measures: CAIC = 736.25 and ECVI = 1.37) were in line with what was statistically required, with values close to and even better than those observed in other studies^(3-6)^.

The guarantee of the adjusted oblique bifactor model, which is capable of measuring sexism mainly in its direct (hostile) and indirect (subtle) forms, probably legitimizes the mechanisms for maintaining gender inequality and targets the relations of dominance between men and women^(5)^. The Ambivalent Sexism Inventory, in the different sample, shows prejudice against women, even though the majority of the sample is made up of women (87.2%).

The bidimensional structure of the inventory portrays a form of modern sexism that not only focuses on hostile aspects, but also represents more subtle aspects of sexism that are justified in apparently egalitarian beliefs. The hostile sexist will most likely be a benevolent sexist. In a cross-cultural study involving 19 countries and more than 15,000 participants, it was observed that the two forms that characterize ambivalent sexism are in fact complementary and constant in different cultures. In general, fluctuations in the levels of hostility and benevolence towards women are correlated with national indices of gender inequality and domestic conventionality^(23-24)^.

Attention is also drawn to the calculation of Cronbach’s alpha (α), one of the most widely used psychometric indicators to verify the internal consistency or validity of the instrument, which obtained 0.86 in the dimension of hostile sexism and 0.82 in benevolent sexism, values that demonstrate the safety of measuring the phenomenon studied in this sample.

Although this research makes contributions to understanding ambivalent sexism in the group studied, it is important to point out its limitations. The sample was restricted to nursing students from a single university, which may limit the generalizability of the results to other populations.

This analysis provides a basis for future application of the instrument in similar contexts, guaranteeing the reliability and validity of the results obtained. By deepening our understanding of the structure and consistency of the ASI, this study could serve as a tool for researchers interested in exploring the phenomenon of ambivalent sexism in different populations and contexts.

The results of this study provide consistent psychometric evidence on the Brazilian version of the ISA, used in a sample of university students from an institution located in the Southeast region of Brazil. This evidence highlights the reliability and validity of the instrument in this context, enabling future investigations into sexism in educational and academic environments.

## Conclusion

The confirmatory factor analysis of the Ambivalent Sexism Inventory in Nursing undergraduates confirmed the adequacy of the bifactor model, composed of the dimensions of hostile and benevolent sexism, with a strong fit to the data. Furthermore, internal reliability coefficients, such as Cronbach’s alpha, together with the Intraclass Correlation Coefficient, reinforce the precision and consistency of the instrument. These results, combined with the construct validity ensured by the bifactor structure, demonstrate that the ISA is a psychometrically solid and reliable tool for assessing sexist attitudes in this population.

The effectiveness of the ISA in detecting different forms of sexism in the academic environment was highlighted, allowing a deeper understanding of the existence of these attitudes among Nursing students. The psychometric consistency of the instrument reinforces its potential as a tool for future investigations into gender issues, especially in fields where gender inequality is present in a subtle and structural way.

We recommend replicating the study with more diverse samples, including teachers, students from different institutions and courses, as well as professionals working in the labor market, in order to gain a more comprehensive understanding of the phenomenon of sexism in nursing. Expanding the research will provide a more holistic view of these issues and enable the development of more effective strategies to tackle sexism and promote gender equality at all levels of the nursing profession.
