# Supplementary material for: Psychometric evidence of the Ambivalent Sexism Inventory in undergraduate nursing students at a public institution
Source: Rev Lat Am Enfermagem. 2025 Jan 31;33:e4413. doi: 10.1590/1518-8345.7179.4413 (PMC11797146; doi:10.1590/1518-8345.7179.4413)
Supplement: Translation

# Translation: Evidencia psicométrica del Inventario de Sexismo Ambivalente en estudiantes de Enfermería de una institución pública^*^

**DOI:** 10.1590/1518-8345.7179.4412

**Authors:** Susane Vasconcelos, Lucia Helena Garcia Penna, Nilton Soares Formiga, Rudson Oliveira Damasceno, Liana Viana Ribeiro, Bianca Carvalho de Abreu Leira

**Affiliations:** 1 Universidade do Estado do Rio de Janeiro, Rio de Janeiro, RJ, Brasil.; 2 Becaria de la Coordenação de Aperfeiçoamento de Pessoal de Nível Superior (CAPES), Brasil.; 3 Universidade Potiguar, Natal, RN, Brasil.; 4 Universidade Estadual do Sudoeste da Bahia, Jequié, BA, Brasil.

**Keywords:** Sexismo, Análisis Factorial, Educación Enfermería, Enfermería, Prejuicios, Derechos de las Mujeres

## Abstract

**Objetivo:**

verificar la consistencia interna y estructural del Inventario de Sexismo Ambivalente en jóvenes del curso de pregrado en Enfermería.

**Método:**

estudio metodológico, de corte transversal, realizado con participantes jóvenes universitarios, matriculados en el curso de pregrado en Enfermería de una universidad pública. Los datos fueron obtenidos mediante cuestionario sociodemográfico/académico e Inventario de Sexismo Ambivalente. El análisis realizado utilizó correlación de Pearson, alfa de Cronbach, correlación intraclase, prueba t y razón chi-cuadrado y grados de libertad, además de análisis factorial confirmatorio para probar la consistencia de la existencia del modelo bifactorial.

**Resultados:**

la muestra estuvo compuesta por 305 estudiantes de pregrado. El modelo bifactorial oblicuo presentó indicadores estadísticos que justifican la consistencia de la estructura bifactorial del sexismo en la población objetivo del estudio. Además, los indicadores psicométricos del inventario revelaron resultados satisfactorios. El análisis de regresión predictiva confirmó la estructura, demostrando su consistencia y robustez para evaluar tanto el sexismo hostil como el sexismo benevolente entre los jóvenes universitarios de Enfermería.

**Conclusión:**

se identificó apoyo a la teoría del sexismo ambivalente, reflejando consistencia del modelo bifactorial oblicuo. El análisis de las propiedades psicométricas del inventario, incluida la validez y confiabilidad, refuerza su aplicabilidad y relevancia en la investigación sobre cuestiones de género en el área de la salud.

## Destacados

**(1)** La escala comprobó la existencia del modelo de sexismo ambivalente en estos estudiantes de pregrado.

**(2)** Presentó puntajes alfa e ICC por encima del estándar psicométrico mínimo exigido.

**(3)** La consistencia interna del inventario demostró mayor precisión en comparación con otros estudios.

**(4)** La estructura multidimensional del inventario refleja una forma de sexismo moderno.

## Introducción

El prejuicio asociado al género sigue siendo un tema actual en el siglo XXI. A pesar del avance científico, se observa que la construcción patriarcal aún promueve la dominación masculina en las relaciones sociales, lo que genera que mujeres con diferentes características sigan siendo objeto de diversas situaciones derivadas de su condición de ser mujer, como la asignación de un papel pasivo, donde el proceso de sumisión femenina es gradualmente considerado legítimo, reforzando los estereotipos relacionados con el sexo y el género construidos socialmente^(1-2)^.

Las visiones estereotipadas sobre mujeres y hombres influyen en la formación y estructuración del sexismo. El término sexismo puede definirse como el conjunto de estereotipos relacionados con la apariencia, los actos, habilidades, emociones y el rol apropiado en la sociedad, en función del género. Aunque también estereotipa al hombre, con frecuencia refleja prejuicios más hostiles hacia el género femenino, manifestándose de manera institucional y en las relaciones interpersonales^(3-4)^. Así, como la definición tradicional de prejuicio, el sexismo se camufla en la sociedad y en las interacciones sociales, sin siempre presentarse de una forma considerada negativa^(2-5)^.

En la Teoría del Sexismo Ambivalente (TSA), desarrollada para comprender la relación entre hombres y mujeres (cisgénero y heterosexuales), presentada por los autores Glick y Fiske en 1996, el sexismo se entiende en dos formas: hostil y benévolo, reflejando de cualquier manera la inferioridad de las mujeres^(6)^. Basados en la teoría, los mismos autores desarrollaron un instrumento capaz de identificar y evaluar estos tipos de sexismo. El llamado Inventario de Sexismo Ambivalente (ISA), utilizado como base en este estudio, busca cuantificar las expresiones del sexismo precisamente en su estructura bifactorial (actitudes sexistas hostiles y benévolas).

Originalmente desarrollado en inglés, el ISA consistía inicialmente en 140 ítems. Tras adaptaciones, una versión reducida de 22 ítems se aplicó en diversas muestras, mostrando coeficientes de consistencia interna aceptables en los estudios. Estos ítems conforman el instrumento final y permiten evaluar los estereotipos asumidos por los géneros masculino y femenino, en relación con las dos dimensiones del sexismo^(6)^.

Los participantes evalúan las afirmaciones en función de su acuerdo o desacuerdo. Los ítems ISA 01, ISA 03, ISA 06, ISA 08, ISA 09, ISA 12, ISA 13, ISA 17, ISA 19, ISA 20 e ISA 22 evalúan creencias benevolentes, como “las mujeres deben ser queridas y protegidas por los hombres” y “el hombre está incompleto sin la mujer”. Mientras tanto, los ítems ISA 02, ISA 04, ISA 05, ISA 07, ISA 10, ISA 11, ISA 14, ISA 15, ISA 16, ISA 18 e ISA 21 exploran creencias hostiles, como “las mujeres intentan ganar poder controlando a los hombres” y “las mujeres feministas están haciendo exigencias completamente irracionales a los hombres”.

Otras escalas e instrumentos han sido creados con el objetivo de identificar y medir el sexismo en diferentes contextos de la sociedad, pero la elección del ISA se debe a su amplia utilización y validación en varios estudios alrededor del mundo, siendo reconocido como una herramienta eficaz para evaluar las actitudes sexistas en distintos contextos^(7-11)^.

El ISA ha revelado indicadores psicométricos con muestras en países como Estados Unidos, Chile, México, Corea, Alemania y Brasil, garantizando su estructura bifactorial atribuida a las actitudes sexistas hostiles y benevolentes^(7)^. En Brasil, el ISA fue adaptado y validado por Formiga, Santos y Gouveia^(5)^. El inventario ha mantenido su calidad psicométrica a lo largo de los últimos años en muestras de hombres, grupos de psicólogos, militares y en la comparación entre brasileños y portugueses^(8-11)^, aunque aún no ha sido utilizado en enfermería.

Para una mejor comprensión, el sexismo puede compararse con un iceberg, donde la mayor parte está oculta, siendo visible solo su punta. El sexismo hostil sería la punta visible del iceberg, la expresión más flagrante y explícita del prejuicio, en su forma tradicional: la mujer es considerada inferior al hombre, incapaz de desempeñar los mismos roles que él. Está marcado por el rechazo, la antipatía y la intolerancia hacia el papel femenino como figura de poder y decisión, menospreciando a las mujeres que desafían los roles conservadores y las ideologías tradicionales de género^(5,12)^.

La parte oculta del iceberg se considera la expresión más moderna del sexismo. De manera benevolente, presenta acciones, aparentemente no prejuiciosas, subjetivamente positivas, que definen a la mujer con una identidad dependiente, frágil, sensible, que necesita apoyo y atención del hombre. También se basa en la negación de la existencia de la discriminación contra la mujer, generando un antagonismo hacia las actuales luchas de las mujeres por una mayor inclusión en la sociedad^(3,13)^.

El sexismo cotidiano también está presente en el entorno académico. La universidad es un espacio de reproducción social, donde ocurre la misma socialización de las relaciones de género que se encuentra “afuera”, es decir, fuera de sus muros. Y, aunque es considerada un espacio de pluralidad de pensamientos, en ella pueden multiplicarse actitudes violentas de discriminación e intolerancia. Además, en este entorno se conservan fuertes estructuras jerárquicas que favorecen las relaciones desiguales^(14)^.

La formación y desarrollo de los estudiantes de enfermería como profesionales dependen de características relacionales y ambientales^(12)^. La enfermería es considerada una profesión históricamente femenina, por sus características y por la prevalencia cuantitativa de mujeres, lo que encuentra en la desigualdad de género un indicador de mayor vulnerabilidad. Las experiencias sexistas, injustas y evitables, deben ser estudiadas para formar mejor a los jóvenes y, en consecuencia, influir positivamente en sus prácticas futuras. La escasez en la producción de estudios en relación con el uso del Inventario de Sexismo Ambivalente en la población de jóvenes estudiantes de enfermería permite que aún existan lagunas en nuestro conocimiento sobre la desigualdad y el sexismo en este contexto. Son necesarios estudios que investiguen y exploren el sexismo en el entorno universitario y en los cursos de enfermería.

De esta manera, este estudio tiene como objetivo verificar la consistencia interna y estructural del Inventario de Sexismo Ambivalente en jóvenes del curso de pregrado en enfermería.

## Método

### Diseño del estudio

Se trata de un estudio metodológico, de corte transversal, con un enfoque cuantitativo, redactado de acuerdo con la herramienta STROBE (*Strengthening the Reporting of Observational Studies in Epidemiology*).

### Escenario del estudio

El estudio se realizó en una Institución de Educación Superior (IES) pública de Rio de Janeiro - RJ, Brasil: *Universidade do Estado do Rio de Janeiro*. La elección de la IES se debió a su reputada trayectoria en el área de enfermería y por ser un centro de investigación reconocido internacionalmente.

### Población y muestra

La población objetivo de este estudio estuvo compuesta por estudiantes de pregrado de enfermería matriculados entre julio y octubre de 2022, totalizando 352 estudiantes. Todos los estudiantes que cumplían con los criterios de inclusión – mayores de 18 años y con matrícula activa – fueron invitados a participar en el estudio. Tras aplicar los criterios de exclusión, que implicaban la no localización del estudiante tras tres intentos, la muestra final estuvo compuesta por 305 participantes. Para determinar el tamaño de la muestra, se tomaron en cuenta las recomendaciones establecidas en la literatura para la realización del análisis factorial confirmatorio, que sugieren la participación de al menos 200 a 300 participantes para asegurar la robustez de las estimaciones de los parámetros del modelo^(15)^. Así, el número de 305 participantes fue considerado adecuado para la realización de los análisis.

### Recolección de datos

La recolección de datos se llevó a cabo entre los meses de junio y octubre de 2022. Los datos fueron obtenidos mediante un instrumento autoaplicable. Inicialmente, la secretaría del curso proporcionó la lista nominal de los estudiantes matriculados por periodos y los horarios de las asignaturas. De esta manera, tras el contacto con el docente responsable de la asignatura seleccionada, se realizaba de forma estandarizada la presentación del estudio y la distribución del instrumento en el aula. Los estudiantes que cumplían con los criterios de inclusión fueron invitados a participar y completar el instrumento de manera individual. Los estudiantes de enfermería tardaron un promedio de 30 minutos en completar las preguntas.

El instrumento de recolección de datos se dividió en dos bloques: el bloque I abordó aspectos sociodemográficos como sexo, edad, estado civil, identidad y expresión de género, orientación sexual, ocupación, religión, ingresos familiares, vivienda y ocio. El bloque II consistió en el Inventario de Sexismo Ambivalente - ISA^(5-6)^.

El inventario, siendo autoaplicable, permitió que el estudiante indicara su nivel de acuerdo con el contenido expresado, utilizando una escala de cuatro puntos, con las siguientes opciones de respuesta: 1 = Totalmente en desacuerdo; 2 = En desacuerdo; 3 = De acuerdo; y 4 = Totalmente de acuerdo.

No se identificaron ítems que generaran dificultades de comprensión por parte de los estudiantes de enfermería durante el proceso de recolección de datos.

### Tratamiento y análisis de los datos

Los datos fueron organizados, tabulados y analizados con el *software Statistical Package for the Social Sciences* (SPSS) versión 25.0. El análisis descriptivo de las características de la población se expresó mediante frecuencias (absoluta y relativa) para las variables categóricas. Para las variables cuantitativas, se utilizaron medidas de tendencia central (media, mediana) y de dispersión (desviación estándar), además de los cálculos relacionados con la correlación de Pearson, alfa de Cronbach (α), correlación intraclase, y razón chi-cuadrado y grados de libertad.

Para verificar la relación del contenido de los 22 ítems del ISA, a partir de su representatividad comportamiento-dominio, se utilizó el análisis correlacional para evaluar la relación teórica presentada en el instrumento desarrollado por los autores antes mencionados, así como las situaciones especificadas en los ítems y cuánto este instrumento representa los aspectos esperados. Para ello, se realizó el cálculo del coeficiente de correlación de Pearson.

Asumiendo teóricamente que el contenido de los ítems del sexismo discrimina y representa el concepto señalado y la propuesta teórico-empírica de la bifactorialidad del constructo (es decir, sexismo ambivalente, estructurado en sexismo benevolente y hostil), se empleó el paquete estadístico *Amos Graphics* 24.0 para el análisis factorial confirmatorio, en el que se hipotetizó el modelo bifactorial observado por los autores mencionados anteriormente. Se esperaba que la organización ítem-factor confirmara una asociación similar a la ya observada.

Se utilizó el análisis factorial confirmatorio para probar la consistencia de la existencia del modelo bifactorial del sexismo (hostil y benevolente) en los estudiantes de pregrado en enfermería. Se decidió dejar libres las covarianzas (*phi*, φ), revelando indicadores de calidad de ajuste para el modelo propuesto cercanos a las recomendaciones de la literatura^(15-16)^.

De esta forma, se consideró como entrada la matriz de covarianzas con el estimador ML (*Maximum Likelihood*). Siendo un tipo de análisis estadístico más riguroso, se buscó evaluar la estructura teórica del ISA, guiándose por las propuestas empíricas y axiomáticas de Glick y Fiske^(6)^ y de Formiga, Santos y Gouveia^(5)^ en relación con la bifactorialidad. En cuanto a este análisis, se consideraron los siguientes indicadores estadísticos para evaluar la calidad factorial: chi-cuadrado/grados de libertad (χ²/gl); raíz cuadrada media residual (RMR); *goodness-of-fit index* (GFI) y *adjusted goodness-of-fit index* (AGFI); *comparative fit index* (CFI); *Tucker-Lewis index* (TLI); *root-mean-square error of approximation* (RMSEA); *expected cross-validation index* (ECVI); *consistent akaike information criterion* (CAIC); *Akaike’s information criterion* (AIC); *Browne-Cudeck criterion* (BCC); *Bayes information criterion* (BIC); cálculo de confiabilidad compuesta (CC) y de la varianza media extraída (VME); alfa de Cronbach (α); criterio de Fornell y Larcker y AVE; coeficiente de correlación intraclase (ICC).

Durante el tratamiento y análisis de los datos, las pruebas estadísticas fueron aplicadas de manera integrada para investigar las propiedades psicométricas de los ítems del ISA. Este enfoque permitió no solo un análisis de la estructura bifactorial del sexismo, conforme a la teoría subyacente al instrumento, sino también validar empíricamente su consistencia.

### Aspectos éticos

Este estudio cumplió con los principios éticos propuestos por la Resolución 674/22 del *Conselho Nacional de Saúde*. Certificado de Presentación para Apreciación Ética (CAAE): 59881522.0.0000.5282.

## Resultados

La población estuvo compuesta por 305 estudiantes, predominando el sexo femenino, con un total de 266 estudiantes, lo que representa el 87,2% de la población. El rango de edad predominante fue entre 20 y 24 años (n=211; 69,2%), con una edad promedio de 23 años (desviación estándar 4,5). Se identificó que el participante más joven tenía 18 años y el más mayor 56 años. En cuanto al color/raza, el 21,0% se identificó como negros (n=64) y el 30,8% como pardos (n=94), mientras que el 47,9% se declaró blanco (n=146). La mayoría de los jóvenes eran solteros (n=281; 92,1%), con un ingreso familiar entre uno y tres salarios mínimos (n=183; 60%).

Para garantizar la calidad de la muestra, se incluyó el criterio de multicolinealidad en la evaluación. Se observó que las correlaciones entre los parámetros de Tabachnick y Fidell presentaron un valor de r ≤ 90, variando entre -0,08 y 0,48, lo que indica la ausencia de variables con un alto grado de correlación, permitiendo así el desarrollo de modelos predictivos y/o correlacionales con bajo margen de error.

Se observa en la Tabla 1 que los estudiantes fueron capaces de identificar y evaluar el sexismo en el contexto verificado, mostrando que todos los ítems fueron significativos en su discriminación. El cálculo de la correlación de Pearson (r) para los 22 ítems reveló correlaciones fuertes y positivas con el constructo general del sexismo (puntuación total), todas ellas significativas. Esto aseguró la dirección conceptual y empírica del constructo, sin excluir ningún ítem de la medida prevista.

El análisis factorial confirmatorio (AFC) reveló que el modelo bifactorial oblicuo (donde los factores se correlacionan) presentó indicadores estadísticos que justifican la consistencia de la estructura bifactorial del sexismo en la población objetivo del estudio, hostil y benevolente, como se muestra en la Tabla 2.

Asimismo, al observar los indicadores estadísticos de comparación que sugieren una evaluación parsimoniosa (AIC, BIC y BCC) del modelo factorial propuesto en relación con el hipotético, se verifica que el modelo bifactorial esperado mostró fuertes indicadores psicométricos, ya que sigue al CAIC y ECVI, destinados a verificar la adecuación del modelo estructural a confirmar (Tabla 3).

**Tabla 1:** - Análisis discriminativo, representatividad de contenido y discriminación de los ítems del Inventario de Sexismo Ambivalente en estudiantes de pregrado de enfermería (n = 305). Rio de Janeiro, RJ, Brasil, 2022

**Variables**	**Mínima**	**Máxima**	**Media**	d.e.*	**Sk** ^†^	**Ku** ^‡^	**Representatividad y discriminación de los ítems**	
							***r*** ^§^ **(≥ 0,50)**	***t*** ^||^ **(≥ 1,96)**
ISA^¶^ 01	1	4	1,34	0,67	2,19	1,63	0,58^§^	-7,68^||^
ISA^¶^ 02	1	4	1,41	0,76	1,88	1,74	0,50^§^	-8,04^||^
ISA^¶^ 03	1	4	1,96	1,01	0,62	0,85	0,50^§^	-8,96^||^
ISA^¶^ 04	1	4	1,50	0,77	1,43	1,22	0,52^§^	-6,33^||^
ISA^¶^ 05	1	4	1,30	0,59	1,09	4,35	0,56^§^	-8,82^||^
ISA^¶^ 06	1	4	1,16	0,43	1,20	1,98	0,58^§^	-6,68^||^
ISA^¶^ 07	1	4	1,25	0,57	1,62	1,29	0,59^§^	-7,61^||^
ISA^¶^ 08	1	4	1,48	0,77	1,55	1,56	0,64^§^	-9,04^||^
ISA^¶^ 09	1	4	1,81	1,01	0,79	0,79	0,62^§^	-10,33^||^
ISA^¶^ 10	1	4	1,25	0,51	1,13	1,53	0,60^§^	-6,95^||^
ISA^¶^ 11	1	4	1,24	0,56	1,74	1,03	0,53^§^	-6,99^||^
ISA^¶^ 12	1	4	1,45	0,75	1,76	1,37	0,66^§^	-10,42^||^
ISA^¶^ 13	1	4	1,29	0,59	1,32	1,65	0,65^§^	-9,07^||^
ISA^¶^ 14	1	4	1,22	0,56	1,90	1,60	0,53^§^	-6,29^||^
ISA^¶^ 15	1	4	1,24	0,55	1,36	1,95	0,67^§^	-7,28^||^
ISA^¶^ 16	1	4	1,23	0,53	1,54	1,65	0,57^§^	-6,59^||^
ISA^¶^ 17	1	4	1,87	1,10	0,85	0,75	0,50^§^	-9,09^||^
ISA^¶^ 18	1	4	1,40	0,78	1,91	1,67	0,53^§^	-8,15^||^
ISA^¶^ 19	1	4	1,98	1,06	0,54	1,11	0,55^§^	-11,02^||^
ISA^¶^ 20	1	4	1,48	0,77	1,62	2,14	0,58^§^	-9,61^||^
ISA^¶^ 21	1	4	1,18	0,48	1,24	1,36	0,56^§^	-7,18^||^
ISA^¶^ 22	1	4	2,09	1,10	0,47	1,19	0,52^§^	-8,77^||^

*d.e. = Desviación estándar; ^†^Sk = Índice de simetría; ^‡^Ku = Índice de Curtosis; ^§^ *r* = Correlación de Pearson (p-valor < 0,01); ^ǁ^ *t =* Test t (p-valor < 0,01); ^¶^ISA = Ítems del Inventario de Sexismo Ambivalente

**Tabla 2:** - Indicadores psicométricos de la estructura factorial del Inventario de Sexismo Ambivalente en estudiantes de pregrado de enfermería (n = 305). Rio de Janeiro, RJ, Brasil, 2022

**Modelos**	**Medidas de ajuste absoluto**				**Medidas de ajuste incremental**			**Medidas de ajuste parsimonioso**	
Estatística	χ²/gl*	RMR^†^	GFI^‡^	AGFI^§^	CFI^‖^	TLI^¶^	RMSEA** (Intervalo)	CAIC^††^	ECVI^‡‡^ (Intervalo)
Modelo1^§§^	2,85	0,06	0,84	0,79	0,84	0,81	0,08 (0,07-0,09)	940,99	2,21 (1,99-2,45)
Modelo2^‖‖^	2,53	0,09	0,88	0,85	0,87	0,84	0,07 (0,06-0,08)	889,09	2,01 (1,81-2,23)
Modelo3^¶¶^	1,51	0,03	0,93	0,91	0,95	0,96	0,04 (0,03-0,05)	736,25	1,37 (1,23-1,53)

*χ²/gl = Relación chi-cuadrado/grados de libertad; ^†^RMR = Raíz cuadrada media residual; ^‡^GFI = *Goodness-of-fit index*; ^§^AGFI = *Adjusted goodness-of-fit index*; ^‖^CFI = *Comparative fit index*; ^¶^TLI = *Tucker-Lewis index*; **RMSEA = *Root-mean-square error of approximation*; ^††^CAIC = *Consistent Akaike information criterion*; ^‡‡^ECVI = *Expected cross-validation index*; ^§§^Modelo1 = Modelo unifactorial; ^‖‖^Modelo2 = Modelo bifactorial ortogonal; ^¶¶^Modelo3 = Modelo bifactorial oblicuo ajustado

**Tabla 3:** - Indicadores psicométricos de parsimonia para la comparación de la estructura factorial-conceptual del Inventario de Sexismo Ambivalente en estudiantes de pregrado de enfermería (n = 305). Rio de Janeiro, RJ, Brasil, 2022

	**Indicador de parsimonia**		
**Modelos**	AIC*	**BIC** ^†^	**BCC** ^‡^
Modelo 1^§^	671,94	883,99	681,27
Modelo2^‖^	610,59	830,09	620,25
Modelo3^¶^	415,27	668,26	426,42

*AIC = *Akaike’s information criteria*; ^†^BIC = *Bayes information criterion*; ^‡^BCC = *Browne-Cudeck criterion*; ^§^Modelo1 = Modelo unifactorial; ^‖^Modelo2 = Modelo bifactorial ortogonal; ^¶^Modelo3 = Modelo bifactorial oblicuo ajustado

Se observa que todas las saturaciones (*Lambdas*, λ) estuvieron dentro del intervalo esperado |0 - 1|, lo que reveló que no hubo problemas en la estimación propuesta de la factorialidad del sexismo (Tabla 4). Además, fueron estadísticamente diferentes de cero (t > 1,96, p < 0,05), lo que confirma la existencia del modelo bifactorial oblicuo, revelando una asociación *Phi* (Φ), positiva y fuerte, entre los factores del sexismo hostil y benevolente, con un valor de 0,68. En esta condición, es probable que el estudiante que presente puntajes más altos en una dimensión también obtenga puntajes altos en la otra dimensión (Tablas 3 y 4).

También se destaca que los indicadores de confiabilidad compuesta (CC) y varianza media extraída (VME), referentes a la validez del constructo, presentaron valores iguales o superiores a los exigidos en la literatura^(15-16)^. Específicamente, para la dimensión de sexismo hostil, los valores de CC y VME fueron 0,90 y 0,50, respectivamente. En cuanto al sexismo benevolente, los valores fueron 0,81 y 0,52. Estos resultados evidencian la confiabilidad y la validez convergente del constructo, demostrando que la estructura bifactorial es adecuada en una muestra de jóvenes estudiantes de enfermería.

Para ofrecer una mayor garantía psicométrica de estos alfas, también se utilizó la correlación intraclase. Considerando esto, se observó que los alfas para las medidas psicológicas utilizadas son consistentes y garantizan la evaluación de los constructos en estos jóvenes. El coeficiente alfa de Cronbach (α) presentó puntuaciones superiores a las esperadas y fue significativo. Es importante señalar los resultados del intervalo de confianza en el coeficiente de correlación intraclase (CCI), los cuales estuvieron en intervalos cercanos a los observados en el alfa de Cronbach (α), lo que garantiza la confiabilidad de la medida en la muestra evaluada.

**Tabla 4:** - Estructura factorial del Inventario de Sexismo Ambivalente en estudiantes de pregrado de enfermería (n = 305). Rio de Janeiro, RJ, Brasil, 2022

ξ*	**χ** ^†^	**λ** ^‡^	**Ɛ** ^§^	**CC** ^‖^	**VME** ^¶^	α**	**ICC** ^††^	**Criterio de Fornell y Larcker**
	SH11	0,70	0,35				0,85 (0,82-0,87)	
	SH15	0,71	0,26					
	SH5	0,69	0,45					
	SH10	0,70	0,49	0,90	0,50	0,86		0,71
	SH16	0,72	0,52					
	SH21	0,75	0,54					
Sexismo hostil (SH)	SH7	0,68	0,46					
⭡	SH4	0,72	0,52					
	SH18	0,77	0,47					
	SH14	0,71	0,37					
	SH2	0,63	0,43					
√^‡‡^= 0,68	SB1	0,63	0,40					
⭣	SB6	0,50	0,25					
	SB12	0,74	0,64					
	SB13	0,70	0,50					
	SB3	0,74	0,16				0,82 (0,79-0,85)	
Sexismo Benévolo (SB)	SB9	0,71	0,38	0,81	0,52	0,82		0,72
	SB17	0,78	0,23					
	SB20	0,66	0,31					
	SB8	0,82	0,38					
	SB19	0,74	0,19					
	SB22	0,66	0,22					

*ξ = Constructo psicológico; ^†^| = Variables (itens); ^‡^λ = Puntajes factoriales de la estructura; ^§^ε = Errores de medición de la estructura; **^‖^** CC = Confiabilidad compuesta; ^¶^VME = Varianza media extraída; **α **=** alfa de Cronbach; ^††^ICC = Correlación intraclase; ^‡‡^√= *Phi*

En la Tabla 5, se observa que la estructura bifactorial fue confirmada con base en las estimaciones predictivas a partir del análisis de regresión. De esta manera, el modelo propuesto, a partir de la identificación de las variables, presentó una razón/criterio que no solo correspondía a lo esperado estadísticamente, sino que también fue diferente de cero (*t*> 1,96, *p*< 0,05), con todas las variables significativas.

**Tabla 5:** - Indicadores de las estimaciones predictivas entre ítems-factores del Inventario de Sexismo Ambivalente en estudiantes de pregrado de enfermería (n = 305). Rio de Janeiro, RJ, Brasil, 2022

**Variables**	**Relación**	**Constructos**	**Estimación**	d.e.*	**Razón/Criterio**	**p-valor**
ISA^†^11	<---^‡^	SH^§^	1,000	---	---	---
ISA^†^15	<---^‡^	SH^§^	1,253	0,121	10,322	0,001
ISA^†^5	<---^‡^	SH^§^	1,051	0,120	8,736	0,001
ISA^†^10	<---^‡^	SH^§^	1,079	0,109	9,922	0,001
ISA^†^16	<---^‡^	SH^§^	0,997	0,110	9,035	0,001
ISA^†^21	<---^‡^	SH^§^	0,838	0,097	8,635	0,001
ISA^†^7	<---^‡^	SH^§^	1,033	0,126	8,192	0,001
ISA^†^4	<---^‡^	SH^§^	0,915	0,146	6,273	0,001
ISA^†^18	<---^‡^	SH^§^	1,225	0,152	8,049	0,001
ISA^†^14	<---^‡^	SH^§^	0,818	0,109	7,475	0,001
ISA^†^2	<---^‡^	SH^§^	1,071	0,149	7,193	0,001
ISA^†^1	<---^‡^	SB^‖^	1,000	---	---	---
ISA^†^6	<---^‡^	SB^‖^	0,486	0,061	7,926	0,001
ISA^†^12	<---^‡^	SB^‖^	1,306	0,117	11,177	0,001
ISA^†^13	<---^‡^	SB^‖^	0,927	0,088	10,527	0,001
ISA^†^3	<---^‡^	SB^‖^	0,823	0,141	5,854	0,001
ISA^†^9	<---^‡^	SB^‖^	1,347	0,161	8,395	0,001
ISA^†^17	<---^‡^	SB^‖^	1,175	0,155	7,585	0,001
ISA^†^20	<---^‡^	SB^‖^	0,949	0,109	8,667	0,001
ISA^†^8	<---^‡^	SB^‖^	0,999	0,112	8,923	0,001
ISA^†^19	<---^‡^	SB^‖^	0,962	0,148	6,492	0,001
ISA^†^22	<---^‡^	SB^‖^	1,065	0,155	6,854	0,001

*d.e. = Desviación estándar; ^†^ISA = Ítems del Inventario de Sexismo Ambivalente; ^‡^<--- = Dirección de la asociación entre el constructo y los ítems del Inventario; ^§^SH = Sexismo hostil; ^‖^SB = Sexismo benevolente

Los análisis estadísticos fueron seleccionados en función de los objetivos del estudio y la naturaleza de los datos recopilados. Los análisis fueron esenciales para evaluar la representatividad comportamiento-dominio de los ítems del instrumento y probar la consistencia del modelo bifactorial del sexismo en los estudiantes de pregrado de enfermería, verificando la adecuación de la estructura teórica del ISA a los datos recopilados.

## Discusión

Los resultados de esta investigación confirman la consistencia interna y estructural del Inventario de Sexismo Ambivalente aplicado a estudiantes de pregrado de Enfermería, respaldando la hipótesis del modelo de dos factores del sexismo (hostil y benevolente) propuesto por los autores Glick y Fiske^(6)^. Estos hallazgos refuerzan la validez del instrumento, al tiempo que amplían la comprensión de la presencia y naturaleza del sexismo hostil y benevolente en esta población. Tal evidencia es fundamental para evaluar cómo se manifiestan las actitudes sexistas en el entorno académico y sus implicaciones para la formación de futuros profesionales.

El inventario desarrollado por los autores^(6)^ ha sido utilizado en otros contextos y poblaciones, presentando una precisión factorial cercana al instrumento original^(8-11,17)^.

El ISA, además de representar adecuadamente el contenido propuesto del sexismo ambivalente, con puntajes alfa e ICC por encima del estándar psicométrico mínimo exigido, demostró consistencia de aplicabilidad en la muestra recolectada. Con estos resultados, es posible destacar que la organización de los ítems del inventario fue coherente con los hallazgos de estudios anteriores, y, en cuanto a la consistencia interna del inventario, los resultados fueron mejores en comparación con estudios previos^(5,18)^.

De este modo, se observó que los estudiantes de enfermería que participaron en el estudio fueron capaces de reconocer el contenido (el sexismo) y el propósito del inventario que se les presentó. Además, cada ítem individual que compone el ASI está relacionado con el inventario en su totalidad. Los resultados psicométricos sugieren que la versión brasileña del ASI evalúa con precisión el sexismo ambivalente, de acuerdo con el constructo utilizado en el modelo teórico del instrumento.

Es consenso que la gran mayoría de las culturas en el mundo forman parte de un sistema patriarcal, que otorga poder y privilegios a la figura masculina, asegurando superioridad y soberanía a los hombres en las relaciones sociales^(2,19)^. Los jóvenes insertados en la sociedad y en este sistema piensan y actúan influenciados por el contexto en el que viven, y aunque no sea intencionado, son influidos y enfrentan las consecuencias del machismo, el prejuicio y las violencias presentes, siendo capaces de identificar situaciones en las que ocurra prejuicio relacionado con el género también en el ámbito académico^(3,20)^.

El sexismo es un tema de estudio constante, principalmente debido a su capacidad de adaptación y camuflaje en la sociedad^(9)^. La manifestación ambivalente del sexismo entre los estudiantes también evidencia un prejuicio hacia las mujeres que, en ciertos momentos, busca ocultarse en situaciones aparentemente no prejuiciosas, probablemente asentándose tempranamente en las actitudes y pensamientos de los individuos. Esto parece confirmar lo obvio dentro del contexto brasileño, que en un primer momento incluso niega la existencia de discriminación contra las mujeres. Sin embargo, en la sutileza, en el elogio, en la idea de protección, amparo y gratitud, se manifiesta el sexismo, y así, entre líneas, legitima las violencias contra las mujeres^(21-22)^.

Además, se observó que los indicadores referentes a las medidas de ajuste absoluto: χ²/gl = 1,51, RMR = 0,03, GFI = 0,93, AGFI = 0,91; medida de ajuste incremental: CFI = 0,95, TLI = 0,96, RMSEA = 0,04 (0,03-0,05); medidas de ajuste parsimonioso: CAIC = 736,25 y ECVI = 1,37) estuvieron de acuerdo con lo exigido estadísticamente, con valores cercanos e incluso mejores que los observados en otros estudios^(3-6)^.

La validación del modelo bifactorial oblicuo ajustado, capaz de medir el sexismo, especialmente en su forma directa (hostil) e indirecta (sutil), probablemente legitima los mecanismos de mantenimiento de la desigualdad de género y promueve las relaciones de dominio de los hombres sobre las mujeres^(5)^. El Inventario de Sexismo Ambivalente, en esta muestra particular, evidencia el prejuicio hacia las mujeres, a pesar de que la mayor parte de la muestra está compuesta por mujeres (87,2%).

La estructura bidimensional del inventario refleja una forma de sexismo moderno que no se centra únicamente en aspectos hostiles, sino que también abarca aspectos más sutiles de sexismo, que se justifican en creencias aparentemente igualitarias. Una persona sexista hostil muy probablemente también será sexista benevolente. En un estudio transcultural que involucró a 19 países y más de 15.000 participantes, se observó que las dos formas que caracterizan el sexismo ambivalente son, de hecho, complementarias y constantes en diferentes culturas. En general, las fluctuaciones en los niveles de hostilidad y benevolencia hacia las mujeres están correlacionadas con los índices nacionales de desigualdad de género y de convencionalismo doméstico^(23-24)^.

También, se destaca el cálculo del alfa de Cronbach (α) como uno de los indicadores psicométricos más utilizados para verificar la consistencia o validez interna del instrumento, obteniendo 0,86 en la dimensión de sexismo hostil y 0,82 en sexismo benevolente, valores que demuestran una fiabilidad sólida para medir el fenómeno estudiado en esta muestra.

Aunque esta investigación aporta contribuciones significativas para la comprensión del sexismo ambivalente en el grupo estudiado, es importante señalar sus limitaciones. La muestra estuvo restringida a estudiantes de enfermería de una sola universidad, lo que puede limitar la generalización de los resultados a otras poblaciones.

Este análisis proporciona una base para la aplicación futura del instrumento en contextos similares, garantizando la confiabilidad y validez de los resultados obtenidos. Al profundizar en la comprensión sobre la estructura y consistencia del ISA, este estudio podrá servir como herramienta para investigadores interesados en explorar el fenómeno del sexismo ambivalente en diferentes poblaciones y contextos.

Los resultados de este estudio proporcionan evidencia psicométrica consistente sobre la versión brasileña del ISA, utilizada en una muestra de estudiantes universitarios de una institución ubicada en la región sureste de Brasil. Esta evidencia resalta la fiabilidad y validez del instrumento en este contexto, permitiendo futuras investigaciones sobre el sexismo en entornos educativos y académicos.

## Conclusión

El análisis factorial confirmatorio del Inventario de Sexismo Ambivalente en estudiantes de grado de Enfermería confirmó la adecuación del modelo bifactorial, compuesto por las dimensiones de sexismo hostil y benevolente, con un fuerte ajuste a los datos. Además, los coeficientes de fiabilidad interna, como el alfa de Cronbach, junto con el Coeficiente de Correlación Intraclase, refuerzan la precisión y consistencia del instrumento. Estos resultados, combinados con la validez de constructo garantizada por la estructura bifactorial, demuestran que el ISA es una herramienta psicométricamente sólida y fiable para evaluar actitudes sexistas en esta población.

La efectividad del ISA en la detección de diferentes formas de sexismo en el entorno académico se destacó, permitiendo una comprensión más profunda de la existencia de estas actitudes entre los estudiantes de Enfermería. La consistencia psicométrica del instrumento refuerza su potencial como herramienta para futuras investigaciones sobre cuestiones de género, especialmente en campos donde la desigualdad de género está presente de manera sutil y estructural.

Se recomienda replicar el estudio en muestras más diversas, incluyendo docentes, estudiantes de diferentes instituciones y cursos, así como profesionales que ya estén en el mercado laboral, para obtener una comprensión más amplia del fenómeno del sexismo en la enfermería. La ampliación de la investigación permitirá una visión más holística de estas cuestiones y posibilitará el desarrollo de estrategias más eficaces para combatir el sexismo y promover la igualdad de género en todos los niveles de la profesión de enfermería.

# Translation: Evidência psicométrica do Inventário de Sexismo Ambivalente em graduandos de Enfermagem de uma instituição pública^*^

**DOI:** 10.1590/1518-8345.7179.4414

**Authors:** Susane Vasconcelos, Lucia Helena Garcia Penna, Nilton Soares Formiga, Rudson Oliveira Damasceno, Liana Viana Ribeiro, Bianca Carvalho de Abreu Leira

**Affiliations:** 1 Universidade do Estado do Rio de Janeiro, Rio de Janeiro, RJ, Brasil.; 2 Bolsista da Coordenação de Aperfeiçoamento de Pessoal de Nível Superior (CAPES), Brasil.; 3 Universidade Potiguar, Natal, RN, Brasil.; 4 Universidade Estadual do Sudoeste da Bahia, Jequié, BA, Brasil.

**Keywords:** Sexismo, Análise Fatorial, Educação em Enfermagem, Enfermagem, Preconceito, Direito das Mulheres

## Abstract

**Objetivo:**

verificar a consistência interna e estrutural do Inventário do Sexismo Ambivalente em jovens do curso de graduação de Enfermagem.

**Método:**

estudo metodológico, de corte transversal, realizado com participantes jovens universitários, matriculados no curso de graduação em Enfermagem de uma universidade pública. Os dados foram obtidos por meio de questionários sociodemográficos/acadêmicos e Inventário de Sexismo Ambivalente. A análise realizada utilizou correlação de Pearson, alfa de Cronbach, correlação intraclasse, teste t e razão qui-quadrado e graus de liberdade, além de análise fatorial confirmatória para testar a consistência da existência do modelo bifatorial.

**Resultados:**

a amostra foi composta por 305 graduandos. O modelo bifatorial oblíquo apresentou indicadores estatísticos que justificam a consistência da estrutura bifatorial do sexismo na população-alvo do estudo. Além disso, os indicadores psicométricos do inventário revelaram resultados satisfatórios. A análise de regressão preditiva confirmou a estrutura, demonstrando sua consistência e robustez para avaliar tanto o sexismo hostil quanto o sexismo benevolente entre os jovens universitários de Enfermagem.

**Conclusão:**

foi identificado suporte à teoria do sexismo ambivalente, refletindo consistência do modelo bifatorial oblíquo. A análise das propriedades psicométricas do inventário, incluindo validade e confiabilidade, reforça sua aplicabilidade e relevância na pesquisa sobre questões de gênero na área da saúde.

## Destaques

**(1)** A escala comprovou a existência do modelo do sexismo ambivalente nestes graduandos.

**(2)** Apresentou escores alfas e ICC acima do padrão psicométrico mínimo exigido.

**(3)** A consistência interna do inventário demonstrou maior precisão quando comparada a outros estudos.

**(4)** A estrutura multidimensional do inventário retrata uma forma de sexismo moderno.

## Introdução

O preconceito associado ao gênero continua a ser uma questão atual no século XXI. Apesar do avanço científico, observa-se que a construção patriarcal ainda produz o cultivo da dominação masculina nas relações sociais, desencadeando que mulheres com diferentes características permaneçam alvo de variadas situações decorrentes da condição de ser mulher, como a atribuição de um papel passivo, em que o processo de submissão feminina é gradualmente considerado legítimo, reforçando os estereótipos relacionados ao sexo e gênero socialmente construídos^(1-2)^.

As visões estereotipadas a respeito de mulheres e homens influenciam na formação e na estruturação do sexismo. O termo “sexismo” pode ser definido como o conjunto de estereótipos relacionados à aparência, aos atos, às habilidades, às emoções e ao papel apropriado na sociedade, que é imposto de acordo com o gênero. Apesar de também estereotipar o homem, o sexismo frequentemente reflete preconceitos mais hostis contra o gênero feminino, manifestando-se de maneira institucional e nas relações interpessoais^(3-4)^. Assim como na definição tradicional de preconceito, o sexismo se camulfla na sociedade e nas interações sociais, nem sempre se apresentando de forma considerada negativa^(2-5)^.

Na Teoria do Sexismo Ambivalente (TSA), desenvolvida para entender a relação entre homens e mulheres (cisgênero e heterossexuais), apresentada pelos autores Glick e Fiske em 1996, o sexismo é entendido de duas formas: hostil e benévolo, refletindo, de toda maneira, a concepção de inferioridade das mulheres^(6)^. Com base na teoria, os mesmos autores desenvolveram um instrumento capaz de identificar e avaliar esses tipos de sexismo. O chamado Inventário do Sexismo Ambivalente (ISA), utilizado como base neste estudo, busca quantificar as expressões do sexismo justamente em sua estrutura bifatorial (atitudes sexistas hostis e benévolas).

Originalmente desenvolvido em inglês, o ISA consistia, inicialmente, em 140 itens. Após adaptações, uma versão reduzida de 22 itens foi aplicada em diversas amostras, demonstrando coeficientes de consistência interna aceitáveis nos estudos. Esses itens compõem o instrumento final e possibilitam a avaliação dos estereótipos assumidos pelos gênero masculino e feminino em relação às duas dimensões do sexismo^(6)^.

Os participantes avaliam as afirmações quanto à concordância ou à discordância. Os itens ISA 01, ISA 03, ISA 06, ISA 08, ISA 09, ISA 12, ISA 13, ISA 17, ISA 19, ISA 20 e ISA 22 avaliam crenças benevolentes, como “as mulheres devem ser queridas e protegidas pelos homens” e “o homem está incompleto sem a mulher”. Enquanto isso, os itens ISA 02, ISA 04, ISA 05, ISA 07, ISA 10, ISA 11, ISA 14, ISA 15, ISA 16, ISA 18 e ISA 21 exploram crenças hostis, como “as mulheres tentam ganhar poder controlando os homens” e “as mulheres feministas estão fazendo exigências completamente irracionais aos homens”^(6)^.

Outras escalas e instrumentos foram criados com o objetivo de identificar e medir o sexismo nos diferentes contextos da sociedade, mas a escolha pelo ISA deu-se por sua ampla utilização e validação em diversos estudos ao redor do mundo, sendo reconhecido como uma ferramenta eficaz para avaliar as atitudes sexistas em diferentes contextos^(7-11)^.

O ISA tem revelado indicadores psicométricos com amostras em países como Estados Unidos, Chile, México, Coreia, Alemanha e Brasil, garantindo a sua estrutura bifatorial atribuída às atitudes sexistas hostis e benevolentes^(7)^. No Brasil, o ISA foi adaptado e validado por Formiga, Santos e Gouveia^(5)^. O inventário vem mantendo a qualidade psicométrica ao longo dos últimos anos em amostras de homens, grupo de psicólogos, militares e na comparação entre brasileiros e portugueses^(8-11)^, porém ainda não é utilizado na enfermagem.

Para melhor entendimento, o sexismo pode ser comparado a um *iceberg,* em que a maior parte fica encoberta, sendo visível apenas a sua ponta. O sexismo hostil seria a ponta visível do *iceberg*, a expressão mais flagrante e explícita do preconceito, em sua forma tradicional: a mulher é considerada inferior ao homem, sendo incapaz de exercer os mesmos papéis que ele. É marcado pela rejeição, antipatia e intolerância em relação ao papel da mulher como figura de poder e decisão, menosprezando as mulheres que desafiam os papéis conservadores e as ideologias tradicionais de gênero^(5,12)^.

A parte encoberta do *icerberg* é considerada a expressão mais moderna do sexismo. De maneira benévola, apresenta ações aparentemente não preconceituosas, subjetivamente positivas, que definem a mulher como uma identidade dependente, frágil, sensível, que necessita de amparo e atenção do homem. Baseia-se também na negação da existência da discriminação contra a mulher, logo, em um antagonismo contra as atuais lutas das mulheres por maior inserção na sociedade^(3,13)^.

O sexismo cotidiano também está presente no ambiente acadêmico. A universidade é um espaço de reprodução social, onde ocorre a mesma socialização das relações de gênero presentes do “lado de fora”, ou seja, fora de suas paredes. E, mesmo sendo considerada um espaço de pluralidade de pensamentos, nela podem multiplicar-se atitudes violentas de discriminação e intolerância. Além disso, nesse meio conservam-se fortes estruturas hierárquicas que favorecem as relações desiguais^(14)^.

A formação e desenvolvimento dos estudantes de enfermagem como profissionais depende de características relacionais e ambientais^(12)^. A enfermagem é considerada uma profissão historicamente feminina por suas características e pelo quantitativo prevalente de mulheres, encontrando na desigualdade de gênero um indicador de maior vulnerabilidade. As experiências sexistas, injustas e evitáveis devem ser estudadas para melhor formar os jovens e, consequentemente, influenciar positivamente suas práticas futuras. A escassez na produção de estudos em relação à utilização do ISA na população de jovens graduandos de enfermagem demonstra que ainda existem lacunas no nosso conhecimento sobre a desigualdade e o sexismo nesse contexto. São necessários estudos que visem pesquisar e explorar o sexismo no meio universitário e nos cursos de enfermagem.

Dessa forma, este estudo tem por objetivo verificar a consistência interna e estrutural do Inventário do Sexismo Ambivalente aplicado em jovens do curso de graduação de enfermagem.

## Método

### Delineamento do estudo

Trata-se de um estudo metodológico, de corte transversal, com uma abordagem quantitativa, redigido de acordo com a ferramenta STROBE (*Strengthening the Reporting of Observational Studies in Epidemiology*).

### Cenário do estudo

O estudo foi realizado em uma Instituição de Ensino Superior (IES) pública do Rio de Janeiro - RJ, Brasil: Universidade do Estado do Rio de Janeiro. A escolha pela IES se deu devido a sua renomada reputação na área de enfermagem e por ser um polo de pesquisa internacionalmente reconhecido.

### População e amostra

A população-alvo deste estudo foi composta por graduandos de enfermagem matriculados entre julho e outubro de 2022, totalizando 352 estudantes. Todos os estudantes que atendiam aos critérios de inclusão – maiores de 18 anos e com matrícula ativa – foram convidados a participar do estudo. Após a aplicação dos critérios de exclusão, que envolviam a não localização do estudante após três tentativas, a amostra final foi composta por 305 participantes. Para determinar o tamanho da amostra, foram consideradas as recomendações preconizadas na literatura para a realização da análise fatorial confirmatória, que sugerem a participação de, pelo menos, 200 a 300 participantes para assegurar a robustez das estimativas dos parâmetros do modelo^(15)^. Assim, o número de 305 participantes foi considerado adequado para a realização das análises.

### Coleta de dados

A coleta foi realizada durante os meses de junho a outubro de 2022. Os dados foram obtidos por meio de instrumento autoaplicável. Inicialmente, a secretaria do curso disponibilizou a lista nominal dos discentes matriculados por períodos e os horários das disciplinas. Dessa forma, após contato com o docente responsável pela disciplina selecionada, realizou-se, de forma padronizada, a apresentação da pesquisa e a distribuição do instrumento em sala de aula. Os alunos que se enquadravam nos critérios de inclusão foram convidados a participar e preencher o instrumento de forma individual. Os discentes de enfermagem levaram em média 30 minutos para concluir o preenchimento das questões.

O instrumento de coleta de dados foi dividido em dois blocos: o bloco I abordou aspectos sociodemográficos como sexo, idade, estado civil, identidade e expressão de gênero, orientação sexual, ocupação, religião, renda familiar, moradia e lazer. Já o bloco II consistiu no Inventário de Sexismo Ambivalente - ISA^(5-6)^.

O inventário, sendo autoaplicável, permitiu que o estudante indicasse o quanto estava de acordo com o conteúdo expresso, utilizando para isso uma escala de quatro pontos com as seguintes possibilidades de respostas: 1 = Discordo totalmente; 2 = Discordo; 3 = Concordo e 4 = Concordo totalmente.

Não foram identificados itens que geraram dificuldades de compreensão por parte dos alunos de enfermagem durante o processo de coleta de dados.

### Tratamento e análise dos dados

Os dados foram organizados, tabulados e analisados no *software Statistical Package for the Social Sciences* (SPSS) versão 25.0. A análise descritiva das características da população foi expressa por meio de frequências (absoluta e relativa) para as variáveis categóricas. Já para as variáveis quantitativas, foram utilizadas medidas de tendência central (média, mediana) e de dispersão (desvio-padrão) e os cálculos referentes à correlação de Pearson, alfa de Cronbach (α), correlação intraclasse e razão qui-quadrado e graus de liberdade.

Na verificação da relação do conteúdo dos 22 itens do ISA, a partir da sua representatividade comportamento-domínio, utilizou-se a análise correlacional para avaliar a relação teórica apresentada no instrumento desenvolvido pelos autores supracitados, bem como as situações especificadas nos itens e o quanto este instrumento representa os aspectos esperados. Para isso, utilizou-se a análise correlacional, realizando o cálculo de coeficiente de correlação de Pearson.

Tendo assumido, teoricamente, que o conteúdo dos itens do sexismo discrimina e representa o referido conceito e a proposta teórico-empírica da bifatorialidade do construto (isto é, seximo ambivalente, estruturado em sexismo benévolo e hostil), empregou-se o pacote estatístico *Amos Graphics* 24.0, destinado à análise fatorial confirmatória para a qual hipotetizou o modelo bifatorial, observado pelos autores supracitados, a partir do qual esperava-se que a organização item-fator confirmasse uma associação semelhante já observada.

Utilizou-se a análise fatorial confirmatória para testar a consistência da existência do modelo bifatorial do sexismo nos alunos do curso de graduação em enfermagem. Optou-se por deixar livres as covariâncias (phi, φ), revelando indicadores de qualidade de ajuste para o modelo proposto próximos às recomendações apresentadas na literatura^(15-16)^.

Dessa forma, considerou-se como entrada a matriz de covariâncias o estimador *Maximum Likelihood* (ML). Sendo um tipo de análise estatística mais criteriosa e rigorosa, buscou-se avaliar a estrutura teórica do ISA, tendo como orientação empírica e axiomática as propostas Glick e Fiske^(6)^ e Formiga, Santos e Gouveia^(5)^, referentes à bifatorialidade. No que se refere a esta análise, foram considerados os seguintes indicadores estatísticos para avaliação da qualidade fatorial: qui-quadrado/graus de liberdade (χ²/gl); raiz quadrada média residual (RMR); *goodness-of-fit index* (GFI) e *adjusted goodness-of-fit index* (AGFI); *comparative fit index* (CFI); *Tucker-Lewis index* (TLI); *root-mean-square error of approximation* (RMSEA); *expectedcross-validation index* (ECVI) e *consistent Akaike information criterion* (CAIC); *Akaike’s information criterion* (AIC); *Browne-Cudeck criterion* (BCC); *Bayes information criterion* (BIC); cálculo de confiabilidade composta (CC) e o da variância média extraída (VME); alfa de Cronbach (α); critério de Fornell e Larcker e variância extraída média (AVE); coeficiente de correlação interclasse (ICC).

Durante o tratamento e análise dos dados, os testes estatísticos foram empregados de maneira integrada para investigar as propriedades psicométricas dos itens do ISA. Essa abordagem permitiu não apenas uma análise da estrutura bifatorial do sexismo, conforme proposto pela teoria subjacente ao instrumento, mas também validou empiricamente sua consistência.

### Aspectos éticos

Este estudo obedeceu aos princípios éticos propostos pela Resolução 674/22 do Conselho Nacional da Saúde. Certificado de Apresentação de Apreciação Ética (CAAE): 59881522.0.0000.5282.

## Resultados

A amostra foi composta por 305 alunos, prevalecendo o sexo feminino, com o total de 266 estudantes, perfazendo 87,2% da população. A faixa etária predominante foi evidenciada entre 20-24 anos (n=211; 69,2%), com média de idade de 23 anos (desvio-padrão de 4,5). Identificou-se que o participante mais novo possuía 18 anos e o mais velho 56 anos. Em relação à cor/raça, 21,0% foram identificados como pretos (n=64), 30,8% como pardos (n=94), e 47,9% declararam-se brancos (n=146). A maioria dos jovens era solteira (n=281; 92,1%), com renda familiar entre um a três salários mínimos (n=183; 60%).

Para garantir a qualidade da amostra, incluiu-se na avaliação o critério da multicolinearidade. Observou-se que as correlações entre os parâmetros de Tabachnick e Fidell tiveram valor de r ≤ 90, variando entre -0,08 e 0,48, indicando a ausência de variáveis com alto grau de correlação, permitindo, assim, o desenvolvimento de modelos preditivos e/ou correlacionais com baixo erro de medida.

Observa-se na Tabela 1 que os alunos foram capazes de identificar e avaliar o sexismo no contexto verificado, mostrando que todos os itens foram significativos na sua discriminação. O cálculo de correlação de Pearson (r) para os 22 itens revelou correlações fortes e positivas com o construto geral do sexismo (pontuação total), todas significativas. Isso garantiu a direção conceitual e empírica do construto, não excluindo qualquer item da medida pretendida.

A análise fatorial confirmatória (AFC) revelou que o modelo bifatorial oblíquo (onde os fatores se correlacionam) apresentou indicadores estatísticos que justificam a consistência da estrutura bifatorial do sexismo na população alvo do estudo, hostil e benévolo, conforme demonstrado na Tabela 2.

Também, ao observar os indicadores estatísticos de comparação que sugerem uma avaliação parcimoniosa (AIC, BIC e BCC) do modelo fatorial pretendido em relação ao de hipótese, verifica-se que o modelo bifatorial esperado teve indicadores psicométricos fortes, pois acompanha o CAIC e ECVI, destinados à verificação da adequabilidade do modelo estrutural a se confirmar (Tabela 3).

**Tabela 1:** - Análise discriminativa, representatividade de conteúdo e discriminação dos itens do Inventário de Sexismo Ambivalente em graduandos de enfermagem (n = 305). Rio de Janeiro, RJ, Brasil, 2022

**Variáveis**	**Mínima**	**Máxima**	**Média**	d.p.*	**Sk** ^†^	**Ku** ^‡^	**Representatividade e discriminação dos itens**	
							***r*** ^§^ **(≥ 0,50)**	***t*** ^||^ **(≥ 1,96)**
ISA^¶^ 01	1	4	1,34	0,67	2,19	1,63	0,58^§^	-7,68^‖^
ISA^¶^ 02	1	4	1,41	0,76	1,88	1,74	0,50^§^	-8,04^‖^
ISA^¶^ 03	1	4	1,96	1,01	0,62	0,85	0,50^§^	-8,96^‖^
ISA^¶^ 04	1	4	1,50	0,77	1,43	1,22	0,52^§^	-6,33^‖^
ISA^¶^ 05	1	4	1,30	0,59	1,09	4,35	0,56^§^	-8,82^‖^
ISA^¶^ 06	1	4	1,16	0,43	1,20	1,98	0,58^§^	-6,68^‖^
ISA^¶^ 07	1	4	1,25	0,57	1,62	1,29	0,59^§^	-7,61^‖^
ISA^¶^ 08	1	4	1,48	0,77	1,55	1,56	0,64^§^	-9,04^‖^
ISA^¶^ 09	1	4	1,81	1,01	0,79	0,79	0,62^§^	-10,33^‖^
ISA^¶^ 10	1	4	1,25	0,51	1,13	1,53	0,60^§^	-6,95^‖^
ISA^¶^ 11	1	4	1,24	0,56	1,74	1,03	0,53^§^	-6,99^‖^
ISA^¶^ 12	1	4	1,45	0,75	1,76	1,37	0,66^§^	-10,42^‖^
ISA^¶^ 13	1	4	1,29	0,59	1,32	1,65	0,65^§^	-9,07^‖^
ISA^¶^ 14	1	4	1,22	0,56	1,90	1,60	0,53^§^	-6,29^‖^
ISA^¶^ 15	1	4	1,24	0,55	1,36	1,95	0,67^§^	-7,28^‖^
ISA^¶^ 16	1	4	1,23	0,53	1,54	1,65	0,57^§^	-6,59^‖^
ISA^¶^ 17	1	4	1,87	1,10	0,85	0,75	0,50^§^	-9,09^‖^
ISA^¶^ 18	1	4	1,40	0,78	1,91	1,67	0,53^§^	-8,15^‖^
ISA^¶^ 19	1	4	1,98	1,06	0,54	1,11	0,55^§^	-11,02^‖^
ISA^¶^ 20	1	4	1,48	0,77	1,62	2,14	0,58^§^	-9,61^‖^
ISA^¶^ 21	1	4	1,18	0,48	1,24	1,36	0,56^§^	-7,18^‖^
ISA^¶^ 22	1	4	2,09	1,10	0,47	1,19	0,52^§^	-8,77^‖^

*d.p. = Desvio-padrão; ^†^Sk = Índice de simetria; ^‡^Ku = Índice de Kurtosis; ^§^ *r* = Correlação de Pearson (p-valor < 0,01); ^||^ *t =* Teste t (p-valor < 0,01); ^¶^ISA = Itens do Inventário de Sexismo Ambivalente

**Tabela 2:** - Indicadores psicométricos da estrutura fatorial do Inventário de Sexismo Ambivalente em graduandos de enfermagem (n = 305). Rio de Janeiro, RJ, Brasil, 2022

**Modelos**	**Medidas de ajuste absoluto**				**Medidas de ajuste incremental**			**Medidas de ajuste parcimonioso**	
Estatística	χ²/gl*	RMR^†^	GFI^‡^	AGFI^§^	CFI^‖^	TLI^¶^	RMSEA** (Intervalo)	CAIC^††^	ECVI^‡‡^ (Intervalo)
Modelo1^§§^	2,85	0,06	0,84	0,79	0,84	0,81	0,08 (0,07-0,09)	940,99	2,21 (1,99-2,45)
Modelo2^‖‖^	2,53	0,09	0,88	0,85	0,87	0,84	0,07 (0,06-0,08)	889,09	2,01 (1,81-2,23)
Modelo3^¶¶^	1,51	0,03	0,93	0,91	0,95	0,96	0,04 (0,03-0,05)	736,25	1,37 (1,23-1,53)

*χ²/gl = Relação qui-quadrado/graus de liberdade; ^†^RMR = Raiz quadrada média residual; ^‡^GFI = *Goodness-of-fit index*; ^§^AGFI = *Adjusted goodness-of-fit index*; ^‖^CFI = *Comparative fit index*; ^¶^TLI = *Tucker-Lewis index*; **RMSEA= *Root-mean-square error of approximation*; ^††^CAIC = *Consistent Akaike information criterion*; ^‡‡^ECVI = *Expected cross-validation index*; ^§§^Modelo1 = Modelo unifatorial; ^‖‖^Modelo2 = Modelo bifatorial ortogonal; ^¶¶^Modelo3 = Modelo bifatorial oblíquo ajustado

**Tabela 3:** - Indicadores psicométricos de parcimônia para a comparação da estrutura fatorial-conceitual do Inventário de Sexismo Ambivalente em graduandos de enfermagem (n = 305). Rio de Janeiro, RJ, Brasil, 2022

	**Indicador de parcimônia**		
**Modelos**	AIC*	**BIC** ^†^	**BCC** ^‡^
Modelo1^§^	671,94	883,99	681,27
Modelo2^‖^	610,59	830,09	620,25
Modelo3^¶^	415,27	668,26	426,42

*AIC = *Akaike’s information criteria*; ^†^BIC = *Bayes information criterion*; ^‡^BCC = *Browne-Cudeck criterion*; ^§^Modelo1 = Modelo unifatorial; ^‖^Modelo2 = Modelo bifatorial ortogonal; ^¶^Modelo3 = Modelo bifatorial oblíquo ajustado

Nota-se que todas as saturações (*Lambdas*, λ) estiveram dentro do intervalo esperado |0 - 1|, o qual revelou não haver problema da estimação proposta da fatorialidade do sexismo (Tabela 4). Além disso, elas foram estatisticamente diferentes de zero (t > 1,96, p < 0,05) comprovando a existência do modelo bifatorial oblíquo, revelando uma associação *Phi* (Φ), positiva e forte, de 0,68, entre os fatores do sexismo hostil e benévolo. Nesta condição, provavelmente, o aluno que apresenta escores mais altos em uma dimensão também pontuará alto na outra dimensão (Tabelas 3 e 4).

Salienta-se também que os indicadores de confiabilidade composta (CC) e variância média extraída (VME), que são referentes à validade do construto, apresentaram valores iguais ou superiores aos exigidos na literatura^(15-16)^. Especificamente, para a dimensão do sexismo hostil, os valores de CC e VME foram de 0,90 e 0,50, respectivamente. Já para o sexismo benevolente, os valores foram de 0,81 e 0,52. Esses resultados evidenciam a confiabilidade e a validade convergente do construto, demonstrando que a estrutura bifatorial é adequada numa amostra de jovens graduandos de enfermagem.

Buscando oferecer maior garantia psicométrica desses alfas, utilizou-se também a correlação intraclasse. Assim considerado, observou-se que os alfas para as medidas psicológicas utilizadas são consistentes e garantem a avaliação dos construtos para esses jovens. O Coeficiente alfa de Cronbach (α) tanto apresentou escores acima do esperado quanto foi significativo. É preciso chamar atenção para os resultados do intervalo de confiança no coeficiente de correlação intraclasse (CCI), os quais estiveram em intervalos próximos aos observados no alfa de Cronbach (α), condição essa que garante a confiabilidade da medida na amostra avaliada.

**Tabela 4:** - Estrutura fatorial do Inventário de Sexismo Ambivalente em graduandos de enfermagem (n = 305). Rio de Janeiro, RJ, Brasil, 2022

ξ*	**χ** ^†^	**λ** ^‡^	**Ɛ** ^§^	**CC** ^‖^	**VME** ^¶^	α**	**ICC** ^††^	**Critério de Fornell e Larcker**
	SH11	0,70	0,35				0,85 (0,82-0,87)	
	SH15	0,71	0,26					
	SH5	0,69	0,45					
	SH10	0,70	0,49	0,90	0,50	0,86		0,71
	SH16	0,72	0,52					
	SH21	0,75	0,54					
Sexismo hostil (SH)	SH7	0,68	0,46					
⭡	SH4	0,72	0,52					
	SH18	0,77	0,47					
	SH14	0,71	0,37					
	SH2	0,63	0,43					
Φ^‡‡^= 0,68	SB1	0,63	0,40					
⭣	SB6	0,50	0,25					
	SB12	0,74	0,64					
	SB13	0,70	0,50					
	SB3	0,74	0,16				0,82 (0,79-0,85)	
Sexismo Benévolo (SB)	SB9	0,71	0,38	0,81	0,52	0,82		0,72
	SB17	0,78	0,23					
	SB20	0,66	0,31					
	SB8	0,82	0,38					
	SB19	0,74	0,19					
	SB22	0,66	0,22					

*ξ = Constructo psicológico; ^†^χ = Variáveis (itens); ^‡^λ = Escores fatoriais da estrutura; ^§^ε = Erros de medida da estrutura; ^‖^CC = Confiabilidade composta; ^¶^VME = Variância média extraída; **α = alfa de Cronbach; ^††^ICC = Correlação intraclasse; ^‡‡^Φ = *Phi*

Na Tabela 5, observa-se o resultado referente à estrutura bifatorial confirmado com base nas estimativas preditivas, a partir da análise de regressão. Sendo assim, o modelo proposto, a partir da identificação das variáveis, apresentou uma razão/critério que não apenas correspondia ao que se esperava estatisticamente, mas que também foi diferente de zero (*t* > 1,96, *p* < 0,05), com todas elas significativas.

**Tabela 5:** - Indicadores das estimativas preditivas entre itens-fatores do Inventário de Sexismo Ambivalente em graduandos de enfermagem (n = 305). Rio de Janeiro, RJ, Brasil, 2022

**Variáveis**	**Relação**	**Construtos**	**Estimativa**	d.p.*	**Razão/critério**	**p-valor**
ISA^†^11	<---^‡^	SH^§^	1,000	---	---	---
ISA^†^15	<---^‡^	SH^§^	1,253	0,121	10,322	0,001
ISA^†^5	<---^‡^	SH^§^	1,051	0,120	8,736	0,001
ISA^†^10	<---^‡^	SH^§^	1,079	0,109	9,922	0,001
ISA^†^16	<---^‡^	SH^§^	0,997	0,110	9,035	0,001
ISA^†^21	<---^‡^	SH^§^	0,838	0,097	8,635	0,001
ISA^†^7	<---^‡^	SH^§^	1,033	0,126	8,192	0,001
ISA^†^4	<---^‡^	SH^§^	0,915	0,146	6,273	0,001
ISA^†^18	<---^‡^	SH^§^	1,225	0,152	8,049	0,001
ISA^†^14	<---^‡^	SH^§^	0,818	0,109	7,475	0,001
ISA^†^2	<---^‡^	SH^§^	1,071	0,149	7,193	0,001
ISA^†^1	<---^‡^	SB^‖^	1,000	---	---	---
ISA^†^6	<---^‡^	SB^‖^	0,486	0,061	7,926	0,001
ISA^†^12	<---^‡^	SB^‖^	1,306	0,117	11,177	0,001
ISA^†^13	<---^‡^	SB^‖^	0,927	0,088	10,527	0,001
ISA^†^3	<---^‡^	SB^‖^	0,823	0,141	5,854	0,001
ISA^†^9	<---^‡^	SB^‖^	1,347	0,161	8,395	0,001
ISA^†^17	<---^‡^	SB^‖^	1,175	0,155	7,585	0,001
ISA^†^20	<---^‡^	SB^‖^	0,949	0,109	8,667	0,001
ISA^†^8	<---^‡^	SB^‖^	0,999	0,112	8,923	0,001
ISA^†^19	<---^‡^	SB^‖^	0,962	0,148	6,492	0,001
ISA^†^22	<---^‡^	SB^‖^	1,065	0,155	6,854	0,001

*d.p. = Desvio-padrão; ^†^ISA = Itens do Inventário de Sexismo Ambivalente; ^‡^<--- = Direção da associação entre o construto e os itens do Inventário; ^§^SH = Sexismo Hostil; ^‖^SB = Sexismo Benevolente

As análises estatísticas foram selecionadas com base nos objetivos do estudo e na natureza dos dados coletados. As análises foram essenciais para avaliar a representatividade do comportamento-domínio dos itens do instrumento e testar a consistência do modelo bifatorial do sexismo nos graduandos de enfermagem, verificando adequação da estrutura teórica do ISA aos dados coletados.

## Discussão

Os resultados desta pesquisa confirmam a consistência interna e estrutural do Inventário de Sexismo Ambivalente aplicado a graduandos de Enfermagem, corroborando a hipótese do modelo bifatorial do sexismo (hostil e benévolo) proposto pelos autores Glick e Fiske^(6)^. Esses achados reforçam a validade do instrumento, ao mesmo tempo em que ampliam a compreensão acerca da presença e natureza do sexismo hostil e benevolente nessa população. Tais evidências são essenciais para avaliar como as atitudes sexistas se manifestam no ambiente acadêmico e suas implicações na formação de futuros profissionais.

O inventário elaborado pelos autores^(6)^ tem sido utilizado em outros contextos e populações, apresentando acurácia fatorial próxima ao instrumento original^(8-11,17)^.

O ISA, além de representar muito bem o conteúdo proposto do sexismo ambivalente, com escores alfas e ICC acima do padrão psicométrico mínimo exigido, apresentou consistência de aplicabilidade na amostra coletada. Com esse resultado, é possível destacar que a organização dos itens do inventário foi correspondente aos já encontrados em estudos anteriores e, em relação à consistência interna do inventário, os resultados foram melhores quando comparados aos encontrados anteriormente^(5,18)^.

Sendo assim, percebeu-se que os estudantes de enfermagem participantes do estudo foram capazes de reconhecer o conteúdo (o sexismo) e o sentido do inventário apresentado a eles. E, ainda, cada item isolado que compõe o ISA se relaciona com o inventário no geral. Os resultados psicométricos sugerem que a versão brasileira do ISA avalia de forma precisa o sexismo ambivalente, segundo o constructo utilizado no modelo teórico do instrumento.

É consenso que a grande maioria das culturas no mundo faz parte de um sistema patriarcal, que concede poder e privilégios à figura masculina, assegurando superioridade e soberania aos homens nas relações sociais^(2,19)^. O jovem inserido na sociedade e nesse sistema pensa e age influenciado pelo contexto em que vive, logo, mesmo que sem intenção, as pessoas são influenciadas e lidam com as consequências do machismo, preconceito e violências presentes, sendo capazes de identificar situações onde ocorra preconceito relacionado ao gênero também no meio acadêmico^(3,20)^.

O sexismo é uma temática que requer estudo, principalmente devido à sua capacidade de adaptação e camuflagem na sociedade^(9)^. A manifestação ambivalente do sexismo também entre os estudantes denuncia um preconceito contra a mulher que, em determinados momentos, busca se disfarçar em situações aparentemente não preconceituosas, que provavelmente se instala precocemente nas atitudes e pensamentos dos indivíduos, o que parece confirmar o óbvio dentro do contexto brasileiro, que, num primeiro momento, até nega a existência da discriminação contra a mulher. Mas, na sutileza, no elogio, na ideia de proteção, amparo e gratidão manifesta o sexismo, e assim, nas entrelinhas, legitimiza as violências contra as mulheres^(21-22)^.

Além disso, observou-se que os indicadores referentes às medidas de ajuste absoluto: χ^.^²/gl = 1,51, RMR = 0,03, GFI = 0,93, AGFI = 0,91; medida de ajuste incremental: CFI = 0,95, TLI = 0,96, RMSEA = 0,04 (0,03-0,05); medidas de ajuste parcimonioso: CAIC = 736,25 e ECVI = 1,37) estiveram de acordo com o exigido estatisticamente, com valores próximos e até melhores aos observados em outros estudos^(3-6)^.

A garantia do modelo bifatorial oblíquo ajustado, capaz de mensurar o sexismo, principalmente, em sua forma direta (hostil) e indireta (sutil) provavelmente legitima os mecanismos de manutenção da desigualdade de gênero e visa às relações de domínio por parte dos homens frente às mulheres^(5)^. O Inventário de Sexismo Ambivalente, na distinta amostra, evidencia o preconceito frente às mulheres, ainda que a maior parte da amostra seja composta por mulheres (87,2%).

A estrutura bidimensional do inventário retrata uma forma de sexismo moderno que não se concentra somente em aspectos hostis, mas também representam aspectos mais sutis de sexismo, que se justificam em crenças aparentemente igualitárias. A pessoa sexista hostil muito provavelmente será sexista benevolente. Em um estudo transcultural, envolvendo 19 países e mais de 15.000 participantes, foi observado que as duas formas que caracterizam o sexismo ambivalente são de fato complementares e constantes em diferentes culturas. De uma forma geral, as flutuações nos níveis de hostilidade e de benevolência, em relação às mulheres, estão correlacionadas com os índices nacionais de desigualdade de gênero e de convencionalismo doméstico^(23-24)^.

Também, chama-se a atenção para o cálculo do alfa de Cronbach (α) quanto ao uso de um dos indicadores psicométricos mais utilizados para verificar a consistência ou validade interna do instrumento, no qual se obteve 0,86 na dimensão do sexismo hostil e 0,82 no sexismo benévolo, valores estes que demonstram uma segurança para medida do fenômeno estudado nessa amostra.

Embora esta investigação apresente contribuições para a compreensão do sexismo ambivalente no grupo estudado, é importante apresentar suas limitações. A amostra foi restrita aos estudantes de enfermagem de uma única universidade, o que pode limitar a generalização dos resultados para outras populações.

Esta análise proporciona uma base para a aplicação futura do intumento em contextos semelhantes, garantindo a confiabilidade e validade dos resultados obtidos. Ao aprofundar a compreensão sobre a estrutura e consistência do ISA, este estudo poderá servir como ferramenta para pesquisadores interessados em explorar o fenômeno do sexismo ambivalente em diferentes populações e contextos.

Os resultados deste estudo fornecem evidências psicométricas consistentes sobre a versão brasileira do ISA, utilizado em uma amostra de estudantes universitários de uma instituição localizada na região Sudeste do Brasil. Essas evidências destacam a confiabilidade e validade do instrumento nesse contexto, possibilitanto futuras investigações sobre sexismo em ambientes educacionais e acadêmicos.

## Conclusão

A análise fatorial confirmatória do Inventário de Sexismo Ambivalente em graduandos de Enfermagem confirmou a adequação do modelo bifatorial, composto pelas dimensões de sexismo hostil e benevolente, com forte ajuste aos dados. Além disso, os coeficientes de confiabilidade interna, como o alfa de Cronbach, juntamente com o Coeficiente de Correlação Intraclasse, reforçam a precisão e consistência do instrumento. Esses resultados, aliados à validade de construto assegurada pela estrutura bifatorial, demonstram que o ISA é uma ferramenta psicometricamente sólida e confiável para avaliar atitudes sexistas nesta população.

A eficácia do ISA em detectar diferentes formas de sexismo no ambiente acadêmico foi evidenciada, permitindo um entendimento mais profundo sobre a existência dessas atitudes entre estudantes de Enfermagem. A consistência psicométrica do instrumento reforça seu potencial como ferramenta para investigações futuras sobre questões de gênero, especialmente em campos onde a desigualdade de gênero se faz presente de forma sutil e estrutural. Recomenda-se replicar o estudo em amostras mais diversificadas, incluindo docentes, estudantes de diferentes instituições e cursos, assim como profissionais atuantes no mercado de trabalho, para uma compreensão mais abrangente do fenômeno do sexismo na enfermagem. A ampliação da pesquisa permitirá uma visão mais holística dessas questões e possibilitará o desenvolvimento de estratégias mais eficazes para enfrentar o sexismo e promover a igualdade de gênero em todos os níveis da profissão de enfermagem.
